# Genome-Wide Association Study for Atopy and Allergic Rhinitis in a Singapore Chinese Population

**DOI:** 10.1371/journal.pone.0019719

**Published:** 2011-05-20

**Authors:** Anand Kumar Andiappan, De Yun Wang, Ramani Anantharaman, Pallavi Nilkanth Parate, Bani Kaur Suri, Hui Qi Low, Yi Li, Wanting Zhao, Paola Castagnoli, Jianjun Liu, Fook Tim Chew

**Affiliations:** 1 Department of Biological Sciences, National University of Singapore, Singapore, Singapore; 2 Department of Otolaryngology, National University of Singapore, Singapore, Singapore; 3 Human Genetics, Genome Institute of Singapore (GIS), Singapore, Singapore; 4 Singapore Immunology Network (SIgN), Agency for Science, Technology and Research (A*STAR), Singapore, Singapore; University of Hong Kong, Hong Kong

## Abstract

Allergic rhinitis (AR) is an atopic disease which affects about 600 million people worldwide and results from a complex interplay between genetic and environmental factors. However genetic association studies on known candidate genes yielded variable results. The aim of this study is to identify the genetic variants that influence predisposition towards allergic rhinitis in an ethnic Chinese population in Singapore using a genome-wide association study (GWAS) approach. A total of 4461 ethnic Chinese volunteers were recruited in Singapore and classified according to their allergic disease status. The GWAS included a discovery stage comparing 515 atopic cases (including 456 AR cases) and 486 non-allergic non-rhinitis (NANR) controls. The top SNPs were then validated in a replication cohort consisting of a separate 2323 atopic cases (including 676 AR cases) and 511 NANR controls. Two SNPs showed consistent association in both discovery and replication phases; *MRPL4* SNP rs8111930 on 19q13.2 (OR = 0.69, P_combined_ = 4.46×10^−05^) and *BCAP* SNP rs505010 on chromosome 10q24.1 (OR = 0.64, P_combined_ = 1.10×10^−04^). In addition, we also replicated multiple associations within known candidates regions such as *HLA-DQ* and *NPSR1* locus in the discovery phase. Our study suggests that MRPL4 and BCAP, key components of the HIF-1α and PI3K/Akt signaling pathways respectively, are two novel candidate genes for atopy and allergic rhinitis. Further study on these molecules and their signaling pathways would help in understanding of the pathogenesis of allergic rhinitis and identification of targets for new therapeutic intervention.

## Introduction

Allergic Rhinitis (AR) represents a global health problem affecting approximately 600 million people in the world population [1], [2]. Though not life threatening the impact of AR on quality of life, school and work performances and productivity is significant [2], [3]. Furthermore it has co-morbidities such as asthma, rhinosinusitis, anosmia, otitis media, nasal polyps and lower airway infection [1], [4], [5], [6]. Atopy is vital in the development of allergic diseases through an IgE-mediated mechanism. It is a genetic predisposition usually starting in childhood or adolescence, when individuals are sensitized and produce IgE antibodies in response to common allergens [7], [8]. However, the mechanism of inheritance, as to how a genetic predisposition leads to allergic symptoms, is still unclear. Many factors have been suggested to play a role in development and expression of atopic diseases such as higher load of irritants and allergen exposure, change in lifestyle, pollution, diet changes with diminished nutritive value and also stress [5], [9], [10], [11]. Thus the pathogenic mechanism of allergic diseases is very complex and is a result of complex interaction between genetic and environmental factors especially in the sensitization phase [1], [4]. The strongest risk factor for the development of allergic symptoms has been a strong family history of allergic disease irrespective of the varying prevalence and environmental risk factors across populations and societies [9], [12]. Various reports support the genetic basis of atopy and allergic disease [13], [14], [15], [16], [17]. Twin studies provide key evidence for a genetic effect as there was a greater concordance of allergic manifestations observed in monozygotic compared to dizygotic twins [18], [19], [20], [21] and the heritability for atopy is estimated to be ranging between 50–84%. Many candidate genes have been suggested for atopy and allergic diseases [7], [8], [19], [22], [23], [24], [25], [26], [27]. To date, a total of five genome wide association (GWA) studies performed to identify loci contributing to the development of asthma and related phenotypes [28], [29], [30], [31]. However, no GWA study has been performed specifically for allergic rhinitis. In this study we carried out two-stage GWAS to identify genetic variants which predispose individuals to the development of atopy and/or allergic rhinitis in Singapore Chinese through a genome wide association study.

## Results

### Discovery phase

The demographics and clinical characteristics of the samples used in the study have been described in **Table 1**. In the Stage 1, 1065 samples (551 cases and 514 controls) were genotyped in 551766 SNPs by using Illumina Human 610 Quad genotyping chip. After stringent quality control filtering for SNPs and samples (see Methods for more details), population stratification was assessed by using an approach based on principal-components analysis (PCA). A total of 25 samples with mixed parentage were identified and removed, and subsequently, PCA for the remaining case and control samples was carried out with a further 3 outliers removed. From the PCA plot, the cases and controls still showed minimal genetic stratification [32]. After all the SNP and sample quality control analyses, genotype data for 460183 SNPs in 515 atopy cases and 486 Non-allergic non-rhinitis (NANR) controls were retained for statistical analysis. A small λ_GC_ value of 1.01 indicated little inflation of the GWAS results due to population stratification.

**Table 1:** Demographic and clinical characteristics of the samples used in the study.

		GWAS		Replication		
	Atopy*	AR$	NANR Controls∧	Atopy*	AR$	NANR Controls∧
**Subjects**	515	456	486	2323	676	511
**Age, mean**	21.3	21.4	21.6	19.99	19.92	19.62
**Gender**						
**Male**	226(43.8%)	213	137(28.2%)	1149(49.5%)	310 (45.86%)	189(36.9%)
**Female**	289(56.2%)	243	349(71.8%)	1174(50.5%)	366(54.14%)	322(63.1%)

* Atopy is defined by a positive SPT reaction to either one of the dust mite allergens (*Dermatophagoides pteronyssinus*, *Blomia tropicalis*).

$ Allergic Rhinitis (AR) was classified based on 2 or more major symptoms which include (nasal congestion, rhinorrhea, nasal itching, sneezing) and a positive skin prick test reaction to one of the allergens tested. *(Based on 2008 guidelines set by Allergic Rhinitis Impact on Asthma (ARIA) consortium)*.

∧ NANR controls are individuals classified based on no symptoms and history of allergic disease and a negative skin prick test reaction to ALL of the allergens tested.

**Figure 1:**
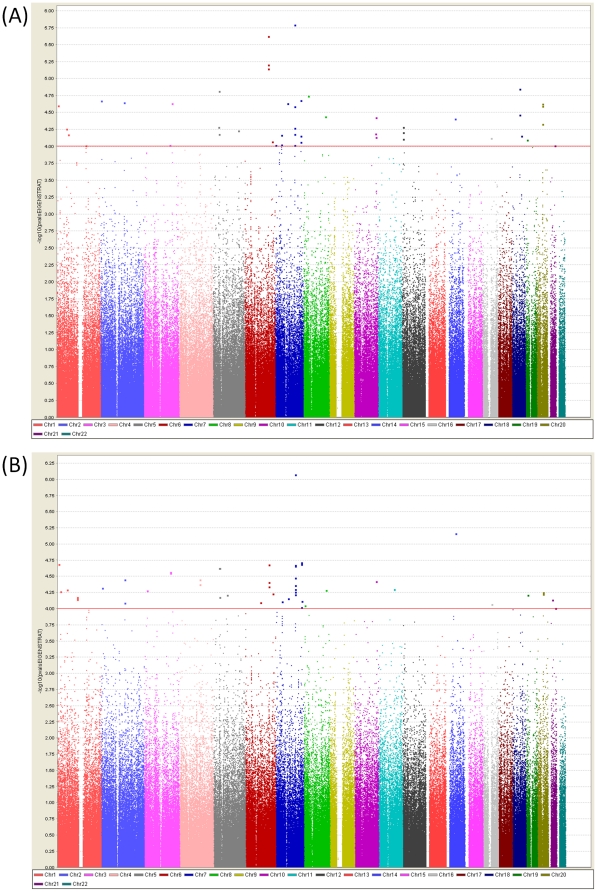
Genome wide association of SNPs for (A) Atopy phenotype (B) AR phenotype.

We then tested for genotype-phenotype association analysis using the Cochran-Armitage trend test. The analysis revealed moderate association at multiple loci throughout the genome (**Figure 1A**). The quantile-quantile (Q-Q) plot of observed *P* values for genome-wide association is shown in **Figure S1**. There were no SNPs which met the genome wide significance threshold (1×10^−4^). However, there were 56 SNPs which showed association with atopy at the significance level of 1×10^−4^. We analyzed the subgroup of Atopic patients with Allergic Rhinitis (AR) clinical symptomology. **Figure 1B** **and Figure S1** shows the results of the genome-wide association study for AR. The GWAS analysis for allergic rhinitis revealed 64 SNPs which had a *P* of less than 1×10^−4^. A total of 31 SNPs were found to be common for both the phenotypes and hence a total of 77 unique SNPs were tested for validation in the replication cohort of 2323 cases and 511 NANR controls, all ethnic Chinese from Singapore (**Tables S1 and S2**).

### Replication Phase and combined analysis

The replication study of atopy identified consistent association at rs8111930 in *MRPL4* (Mitochondrial ribosomal protein L4) gene (OR = 0.78, *P* = 0.029) and rs505010 located in the 5′ upstream region of the *BCAP* (B cell adaptor for phosphatidylinositol 3-kinase) gene (OR = 0.74, *P* = 0.06) (**Table 2**). The Q-Q plot of the p values of the 77 SNPs does not provide evidence for excess associations in the validation samples for the atopy phenotype (**Figure S4A**). Interestingly, the QQ plot of the 77 SNPs in the validation samples of AR (**Figure S4B**) does show a clear deviation from the null, suggesting that there are likely some true associations within the 77 SNPs. However, none of the associations for AR reached the genome-wide significance due to the limited sample size of the study. The joint analysis of the combined GWAS and validation samples reveal suggestive associations at the two SNPs (rs8111930, OR = 0.69, *P*_combined_ = 7.92×10^−5^; rs505010, OR = 0.64, *P*_combined_ = 4.09×10^−04^), however both of them failed to reach genome-wide significance levels. Furthermore, the replication study of allergic rhinitis also revealed consistent association at rs8111930 (OR = 0.76, *P* = 0.049) and rs505010 (OR = 0.63, *P* = 0.027) (**Table 3**). The combined analysis revealed similar association for the atopy phenotype (rs8111930, OR = 0.54, *P*_combined_ = 1.34×10^−04^; rs505010, OR = 0.64, *P*_combined_ = 6.61×−10^−05^), but the evidence for this association is only suggestive. Furthermore, suggestive associations were also observed at 2 other SNPs, rs13188584 in *CSF1R* gene (OR = 1.46, *P*_combined_ = 7.56×10^−05^) and rs10493377 in the 5′ or 3′flanking region of *DNAJC6* (OR = 1.35, *P*_combined_ = 9.50×10^−04^).

**Table 2:** Summary of results of SNPs significant in the validation study for Atopy phenotype.

			GWAS				Replication		GWAS+Replication(2838 cases vs 997 controls)	
CHR	SNP	Gene	Minorallele	Majorallele	*P_trend_*	OR (L95 – U95)	*P_trend_*	OR (L95 – U95)	*P_combined_*	OR
19	rs8111930	MRPL4	A	G	8.69E-05	0.50 (0.35–0.71)	2.98E-02*	0.78 (0.62–0.98)	4.46E-05	0.69 (0.57–0.82)
10	rs505010	BCAP (5′utr)	C	T	1.19E-04	0.40 (0.25–0.64)	6.09E-02	0.74 (0.55–1.01)	1.10E-04	0.61 (0.48–0.79)

**Table 3:** Summary of results of SNPs significant in the validation study for AR phenotype.

			GWAS				Replication		GWAS+Replication(1132 cases vs 997 controls)	
CHR	SNP	Gene	Minorallele	Majorallele	*P_trend_*	OR (L95 – U95)	*P_trend_*	OR (L95 – U95)	*P_combined_*	OR
10	rs505010	BCAP (5′utr)	C	T	1.11E-03	0.44 (0.27–0.72)	2.69E-02*	0.63 (0.42–0.95)	1.34E-04	0.55 (0.40–0.74)
19	rs8111930	MRPL4	A	G	1.25E-04	0.49 (0.34–0.71)	4.95E-02*	0.76 (0.57–1.0)	7.26E-05	0.64 (0.52–0.80)
5	rs13188584	CSF1R	T	C	2.29E-04	1.62 (1.25–2.10)	7.77E-02	1.27 (0.97–1.66)	9.95E-05	1.44 (1.20–1.73)
1	rs10493377	DNAJC6 (3′utr)	A	G	1.52E-03	1.54 (1.18–2.01)	9.52E-02	1.22 (0.97–1.55)	7.85E-04	1.35 (1.14–1.62)

*P_trend_* - *P* values calculated using Cochran Armitage Trend test;

*P value of association <0.05 in the replication study;

*P_combined_* - *P* values calculated combining results from both the GWAS discovery and validation phase using logistic regression.

### Imputation Analysis

**Figure 2:**
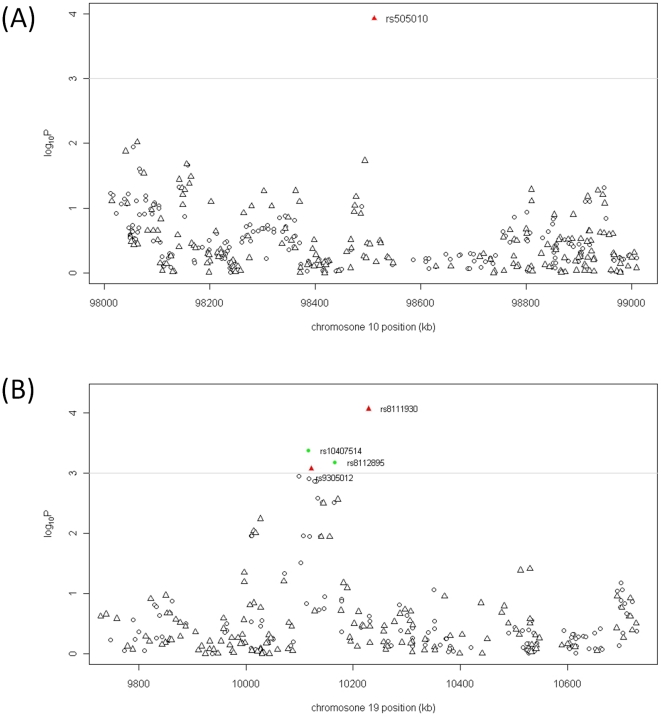
Regional plots of association results within two susceptibility loci. Association results of both genotyped (triangles) and imputed (circles) SNPs in the GWAS samples for the regions of ∼1000 kb containing rs505010 and rs8111930. For each plot, the −log10 P values (y axis) of the SNPs are presented according to their chromosomal positions (x axis), with a grey horizontal line included to indicate suggestive genome-wide significance (10–3). The genotyped and imputed SNPs are indicated by red-triangles and green-circles respectively. The top SNP is labeled by rs ID.

To further investigate the observed associations, we imputed the genotypes of additional SNPs within the regions surrounding rs8111930 and rs505010 that were not genotyped by using IMPUTE (v2.0) and the haplotype information from the HapMap CHB and CHD samples. Only the imputed genotypes with a posterior probability score of >0.90 were used for association analyses. The regional plots for the two regions have been shown in **Figure 2A, 2B**, and the imputed SNPs, however, did not reveal any stronger association than the genotyped SNPs.

### Bioinformatics analysis of validated SNPs for putative TFBS

The SNPs which were validated in the replication population were analyzed for potential transcription factor binding sites (TFBS) using TRANSFAC [33] and MATCH [34] and verified using ALIBABA 2.1 computer program [Available at http://www.gene-regulation.com/pub/programs/alibaba2/index.html. Accessed 2010 Nov 15]. The results have been summarized in **Table S3**. Information about the transcription factors potentially binding to the validated SNPs has been summarized in **Table S4**. Bioinformatics analysis predicts that the introduction of the SNP rs505010 (C→T) results in the loss of transcription factor binding sites (TFBS) for 2 important transcription factors (TF), NFAT (Nuclear factor of activated T-cells) and PU (Ets-like transcription factor identified in lymphoid B-cells). Interestingly this polymorphism results in the introduction of a new TFBS for E2F, which is involved in cell cycle regulation, interacts with Rbp107 protein. The SNP rs8111930 (A→G) results in the loss of TFBS for AREB6 (Atp1a1 regulatory element binding factor 6) and introduces a new TFBS for CREB2 (cAMP-responsive element binding protein).

### Non-synonymous SNPs and the predicted function of the associated SNPs

We also evaluated the associations of non-synonymous SNPs to atopy and allergic rhinitis separately, and the SNPs with at *P* values<0.01 has been described in **Table S6**. The Q-Q plot for these SNPs (**Figure S5**), however, does not provide strong evidence for excess associations among these SNPs. The SNPs, rs273957 (*CREB3L2*), rs2472553 (*CHRNA2*), rs897945 (*THAP9*), rs625372 (*SN*), rs1919127 (*C2orf16*) with *P* values <0.001 were subjected to further evaluation for putative effects on the amino acid and in turn the protein. **Tables S7 and S8** describe the putative effect of these SNPs using the SIFT algorithm and the FASTSNP algorithm. The SIFT algorithm describes whether the change in amino acid could be “tolerated” or may be damaging for the protein. Similarly FASTSNP uses a SNP prioritization ranking and gives a risk score for each SNP also considering conserved regions. Thus the effect of the SNP could be estimated based on the risk score. But further replication in other population cohorts and also functional studies are needed to validate if these SNPs actually have an effect on the disease.

## Discussion

This is the first GWAS study for AR. We had carried out the study in two phases: a discovery phase which consisted of 515 atopic cases including 456 AR cases and 486 NANR controls with no medical history of allergy and a validation phase of 2323 atopic cases including 676 AR cases and 511 NANR controls. No associations were discovered at genome-wide significance; however we observed suggestive associations at rs8111930, an intronic SNP on *MRPL4* gene on chromosome 19p13.2 and rs505010 a 5′ flanking SNP to *BCAP* gene on chromosome 10q24.1 which showed consistent association evidence between the discovery and validation samples.

The 19p13.2 locus (rs8111930) was previously reported to be associated to inflammatory adhesion process and also influencing soluble ICAM1 (sICAM1) levels [35] and sICAM1 has been reported by multiple studies to be a key regulator of nasal allergic reaction [36]. The SNP rs8111930 is an intronic SNP within *MRPL4* gene that codes for the 39S mitochondrial ribosomal protein L4 (**Figure S2**). The protein is believed to play a role in maintaining the structural integrity of the ribosome and also in mitochondrial protein translation. Interestingly, transcription of the *MRPL4* gene is shown to be down-regulated in TGF-β differentiated cells [37]. Since MRPL4 is an important component of the mitochondrial machinery, its regulation by various transcription factors might be vital in understanding how this gene might be involved the allergic inflammation pathway. *In silico* analysis of rs8111930 (A→G) results in the loss of TFBS for AREB6 (Atp1a1 regulatory element binding factor 6) and introduces a new TFBS for CREB2 (cAMP-responsive element binding protein). AREB6 is a negative regulator of IL-2 gene transcription after activation of T-cells and is also suggested to be involved in tissue-specific gene expression and also in early development [38], [39]. Thus studying the regulation of the *MRPL4* gene by its polymorphisms might reveal important clues how they predispose and/or moderate allergic inflammation.

SNP rs505010 is present in the 5′ flanking region of *BCAP* gene (**Figure S3**). BCAP is cytosolic adaptor that bridges the B cell receptor associated kinases to phosphatidylinositol 3-kinase (PI3K) pathway by regulating the localization of PI3K [40], [41]. It is also involved in the activation, development, and maturation of B cells and recent reports have shown their role in activation in natural killer (NK) cells [42], [43]. BCAP is demonstrated to be complimentary in function to CD19 in PI3K activation and suggested to have an important immunoregulatory role in the survival of mature B cells via activation of c-Rel [41], [44]. BCAP-deficient mice have a considerably lower number of mature B cells and whose expansion is compromised on BCR stimulation [41], [43]. This impaired function in mice lacking BCAP results in a loss of function phenotype for B cells [43], [45]. In contrast NK cells from mice deficient for BCAP are considerably more long lived, resistant to apoptosis and have a more mature phenotype with increased functional activity and enhanced cytokine production compared to natural killer cells from normal wild type mice [43]. Mutant mouse models of other signaling molecules such as PLC gamma 2, Btk, Vav, and p85 alpha subunit of PI3K have also resulted in reduced B cell development [41], [43], [46], [47]. However the NK cells of these mutant mice are hypo-responsive, contrary to the NK cells from BCAP-deficient mice. Hence therapeutic manipulation of BCAP to expand development and function of NK cells while promoting B cell apoptosis would help in developing strategies to treat diseases [43], [45]. *In silico* analysis of the associated SNP, predicts that the SNP rs505010(C→T) results in the loss of transcription factor binding sites (TFBS) for 2 important transcription factors (TF), NFAT (Nuclear factor of activated T-cells) and PU (Ets-like transcription factor identified in lymphoid B-cells). Nuclear factor of activated T cells (NFAT) is a T-cell-specific transcription factor which enhances the transcriptional activation of GATA3 by targeting the IL-4 promoter [48]. Recently various studies have also suggested that NFAT inhibitors in models of allergic disease will help understand the efficacy of the calcineurin inhibitors which are currently being tested in the clinic [49]. PU.1 strikingly modulates the levels of TCR expression in CD4 (+) T cells by regulating the DNA-binding activity of GATA-3 [50]. PU.1 has been also shown to be a key regulator of transcription of the *cathepsin G* gene, which has been associated to allergic rhinitis previously [51]. Interestingly this polymorphism results in the introduction of a new TFBS for E2F, which is involved in cell cycle regulation and also interacts with Rbp107 protein. Thus *BCAP* gene polymorphisms could be significant in allergic predisposition and progression.

HIF-1α has been reported to be playing significant roles in inflammatory responses and its inhibition results in reduced bronchial hyper responsiveness [52]. The PTEN/PI3K pathway has been targeted towards treatment for asthma and other allergic phenotypes [53]. Interestingly in a murine model of allergic airway diseases, mast cells have shown to regulate the activity of HIF-1α by a PI3K/Akt signaling pathway [54], [55]. They also demonstrated that increased PI3K activity resulted in higher HIF-1α levels, which were reduced on treatment with inhibitors of PI3K. These results suggest that HIF-1α is a one of the downstream targets of PI3K. However, MRPL4 has been recently identified to be a downstream target of HIF-1α by functional pathway analysis [56]. Hence the study of polymorphisms in *MRPL4*, *BCAP* and other molecules involved in the HIF-1α and PI3K/Akt signaling pathways might help understand how they are regulated and in turn shed light on the pathophysiology of allergic rhinitis and other atopic phenotypes. Taken together these data suggest that pathway controlling signaling of HIF-1α would interact with molecules in the PI3K signaling pathways which might lead to the development and progression of allergic phenotypes such as atopy and allergic rhinitis. Hence the study of MRPL4, BCAP other molecules involved in the HIF-1α and PI3K/Akt signaling pathways might help understand the pathophysiology of allergic rhinitis and other atopic phenotypes. Recent research has also shown promise of therapeutic intervention of the HIF-1α as well PI3K/Akt signaling pathways for treatment of asthma and other related allergic conditions [57], [58], [59].

The study does have its limitations. The first limitation is the sample size for the GWAS and the replication study. The power calculation for Minor allele frequencies (MAF) thresholds and different effect sizes based on Odds Ratio has been summarized in **Table S9**. This was calculated using the CaTS power calculator [http://www.sph.umich.edu/csg/abecasis/CaTS/reference.html. Accessed 2011 April 6]. There was no SNP which met the genome-wide significance and none of the replication SNPs met the Bonferroni correction threshold for multiple testing. Another limitation is that we only selected 77 SNPs (0.00015% of SNPs genotyped in the GWAS phase) to attempt for replication. Through our findings in this study, we suggest that it is unlikely to have any genetic risk factors with effect sizes of OR>1.8 which would have been observed otherwise. However it would be interesting to use the data available to perform meta-analysis or replication in other population in future studies to evaluate the significance of our results.

Interestingly, evaluation of SNPs in candidate genes/regions previously reported in GWAS studies for asthma related traits revealed a strong overlap between atopy, allergic rhinitis and asthma as the QQ plots showed a significant deviation from null (**Figure S6**). Taking a closer look at the SNPs in **Table S5**, it is evident that genes such as *HLA-DQB1*, *HLA-DRB1*, *HLA-DQA2*, *NPSR1* and *PIP-3E* are indeed functionally significant in allergy and related phenotypes. Hence the overlap among the various phenotypes is quite justified in light of their role in the central pathogenesis of the allergic phenotype.

In summary using a cohort of 4461 ethnic Chinese from Singapore we performed the GWAS for atopy and AR and suggested 2 novel susceptibility loci for atopy and allergic rhinitis. Further studies would help to confirm and elucidate the role of these loci in relation to allergic phenotypes. Some of the polymorphisms previously identified through GWAS for asthma and related phenotypes also showed suggestive associations in our GWAS samples, including the SNPs within previously known candidate regions such the *HLA* locus on chromosome 6 and the *NPSR1* locus. Hence these results suggest that genetic susceptibility to complex diseases such as atopy, allergic rhinitis and allergic asthma might involve a large number of genetic variations, ranging between rare alleles with strong effects to intermediate to common alleles with small to moderate effect sizes [60]. Hence further studies with bigger samples and also the functional characterization of these disease associated variants would help elucidate the complex mechanism underlying the genetic predisposition of diseases.

## Materials and Methods

### Ethics Statement

This study has been performed with the approval of the Institutional Review Board of National University of Singapore (IRB, Reference - NUS07-023 and NUS10-343) and is also in compliance with the Helsinki declaration. DNA samples used in this study were collected from ethnic Chinese participants following standard protocols of informed consent. The consent obtained was a “written consent” collected using the Participant Information Sheet which had information about the study.

### Study subjects

A total of 4461 study subjects were recruited from the Singapore Chinese ethnicity through multiple volunteer recruitments in Singapore. Study subjects were subsequently classified as atopy cases, AR cases and non-atopic and non-rhinitis (NANR) healthy controls according to their disease status as determined by ARIA document [1], [4] based questionnaires. Diagnosing procedure included interview of medical history using a standardized questionnaire and skin prick test (SPT) using a panel consisted of common allergens in Singapore such as *Dermatophagoides pteronyssinus*, *Blomia tropicalis*, *Elaeis guineensis* and *Curvularia lunata*. A wheal diameter of 3 mm or greater is considered as a positive SPT response. A positive control with histamine and a negative control of saline were always used. SPT was performed on the volunteers only if they have not taken any anti-allergic drugs especially antihistamines for at least 3 days prior to the test. Atopy is defined by a positive SPT reaction to either one of the dust mite allergens. AR is thus diagnosed based on the presence of atopic status and typical AR symptoms as defined by the ARIA guidelines [1], [4] i.e., two or more AR symptoms (nasal congestion, rhinorrhea, nasal itching, sneezing) persisting for four or more days a week during the past year. Conversely, the subjects with NANR are confirmed with no atopy and no typical AR symptoms. Subjects who could not be recruited into any of the above categories were excluded from study.

Genomic DNA was extracted from buccal cells obtained from a mouthwash of 0.9% saline solution following an in-house standardized protocol. In short, the buccal cells were pelleted and lysed; DNA was extracted using the phenol-chloroform phase-separation technique purified by two washes in ethanol, with the DNA pellet resuspended in reduced Tris-EDTA buffer [61]. Samples were quantified in triplicate on the Nanodrop (ND-1000). Atopy (n = 2849) is defined by a positive SPT reaction to either one of the dust mite allergens. AR (n = 1142) is thus diagnosed based on the presence of atopic status and typical AR symptoms as defined by the ARIA 2008 guidelines [1], [4] i.e., two or more AR symptoms (nasal congestion, rhinorrhea, nasal itching, sneezing) persisting for four or more days a week and lasting for more than 4 weeks continuously. Conversely, the subjects with NANR (n = 1013) are confirmed with no atopy and no typical AR symptoms. A total of 599 subjects who could not be diagnosed into any of the above categories or those whose DNA samples didn't pass the QC criteria were excluded from study.

### Genotyping

A two-stage design for this study: an initial GWAS screening (the discovery phase) and a follow-up (the replication phase) analysis was performed.

### (1) Discovery phase

A total of 1065 unrelated samples were genotyped using the Illumina HumanHap 550 k BeadChip, version 3 (Illumina, San Diego, California) at the Genome Institute of Singapore, Genotyping core facility. Genotypes were determined using the Illumina Genome Studio Module, following recommended protocol. As a part of quality control, SNPs were excluded if they showed either a call rate lower than 98% in cases or controls, a minor allele frequency <1% in the population or significant deviation from the Hardy-Weinberg equilibrium (HWE) in the controls with HWE *P* value <10^−7.^ Furthermore, all SNPs on the X, Y and mitochondrial chromosomes as well as the CNV-related SNPs and probes were excluded from statistical analysis.

### (2) Replication phase

Genotyping of new additional 2834 samples for SNPs selected for replication were performed using matrix assisted laser desorption/ionization time of flight mass spectrometry (MALDI-TOF MS) for the determination of allele specific primer extension products using Sequenom's MassARRAY system and iPLEX technology (Sequenom Inc, San Diego). The design of oligonucleotides was carried out according to the guidelines of Sequenom and performed using MassARRAY Assay Design software. Multiplex PCR amplification of amplicons containing SNPs of interest was performed using Qiagen HotStart Taq Polymerase using 5 ng of genomic DNA. Primer extension reactions were carried out according to manufacturer's instructions for iPLEX chemistry. Assay data were analyzed using the Sequenom TYPER software. Clustering of genotype calls was evaluated to determine that the clustering was sufficient for inclusion in the statistical analysis. All SNPs were also checked for Hardy-Weinberg equilibrium (HWE) and SNPs were not analyzed further if they failed HWE.

### Statistical Analysis

All statistical analyses were performed by using R program version 2.10.1 and PLINK version 1.05. As a part of quality control, we examined the potential genetic relatedness based on pairwise identity by state for all the successfully genotyped samples using the PLINK v1.05 software. For any pair of samples identified as first degree relatives, the sample with lower SNP call rate was removed. The remaining samples were subsequently assessed for all population outlier and stratification using a principal component analysis (PCA) based approach [62]. The original script from EIGENSTRAT was modified so as to extract the principal component analysis and plot them. Firstly, PCA was used to identify genetic outlier using all samples which were genotyped successfully and passed the QC as mentioned above along with the Hapmap samples. The Hapmap reference samples were drawn from the International Hapmap Project: 57 Yoruba in Ibadan, Nigeria (YRI), 44 Japanese in Tokyo, Japan (JPT), 45 Han Chinese in Beijing, China (CHB) and 60 CEPH (Utah residents with ancestry from northern and western Europe) (CEU). We employed very stringent criterion for the removal of the genetic outliers. A total of 25 samples were removed with first principal component less than 0.001 and fourth principal component greater than 0.1 were removed from the genome-wide analysis. Second, PCA was used to assess if population stratification exists in our case and control samples individually. Two outlier samples (both cases) were removed and a genomic control of λ_GC_ = 1.0132 was applied to the association analysis to account for the minimal population stratification that existed. We checked MAF of the remaining samples again and14763 SNPs with MAF <1% were further removed. After all the SNP and sample quality control analyses, genotype data of 460183 SNPs in 515 cases and 486 NANR controls in Singapore population were used in the GWAS. We performed genome-wide association analyses of Atopy and AR in a set of 515 Atopy cases (456 cases with AR) and 486 NANR controls.

We performed genotype-phenotype association analysis using the Cochran-Armitage trend test with PCA-based correction for population stratification and calculated odds ratio (OR) per allele. We used the quantile-quantile (Q-Q) plot to evaluate the overall significance of the genome-wide association results and the potential impact of population stratification. The impact of population stratification was also evaluated by calculating the genomic control inflation factor (λ_GC_). There was minimal population stratification reflected by a λ_GC_ = 1.0132 which was accounted for while calculating the association results. Similar analysis for the AR phenotype consisting of 456 AR cases and 486 NANR controls revealed a λ_GC_ = 1.001. Association analysis for the SNPs genotyped in the replication phase were carried out using the PLINK v1.05 software. All p-values from the replication analysis were reported without correction for multiple testing. Association analysis of the combined samples from the discovery phase and replication phase samples was done using the Cochran-Mantel-Hanezel stratification analysis.

### Imputation

Untyped genotypes were imputed in the GWAS samples by using IMPUTE (v2.0) and the haplotype information from the Hapmap CHB and CHD samples. As part of the quality control, SNPs with a call rate <90%, MAF <0.01 or significant deviation from Hardy-Weinberg Equilibrium in the controls (7*10-5) were removed. The association test was performed using a logistic regression analysis adjusted for study and population stratification of GWAS samples as described above. Regional plots were generated using R to show the −log10 P values.

## Supporting Information

Table S1 Summary of results of SNPs selected for validation in the replication population for Atopy phenotype.
(DOC)

Table S2 Summary of results of SNPs selected for validation in the replication population for AR phenotype.
(DOC)

Table S3 Predicted effect of SNPs validated in replication study on Transcription Factor Binding Sites.
(DOC)

Table S4 Further information on Transcription Factor Binding Sites (TFBS).
(DOC)

Table S5 *P*-values of SNPs from previous GWAS reported loci for asthma phenotype in Singapore Chinese GWAS.
(DOC)

Table S6 Non-synonymous SNPs from the Illumina 550 k chip at a *P*-value<0.01.
(DOCX)

Table S7 Putative effect of the non-synonymous SNPs as predicted by SIFT.
(DOCX)

Table S8 Putative function of non-synonymous SNPs.
(DOCX)

Table S9 Power calculation for one stage design to detect association at genome wide significance (*P*<5*10^−8^).
(DOCX)

Figure S1 Quantile-quantile (Q-Q) plots of the observed *P* values versus the expected values from *P* value of association for (A) Atopy and (B) Allergic Rhinitis.
(DOC)

Figure S2 Genomic organization of a 200-kb region of chromosome 19p13.2 containing rs8111930 with linkage disequilibrium information.
(PDF)

Figure S3 Genomic organization of a 200-kb region of chromosome 10q24.1 containing rs505010 with linkage disequilibrium information.
(PDF)

Figure S4 Quantile-quantile (Q-Q) plots of the observed *P* values versus the expected values from *P* value of association for the replication SNPs tested for (A) Atopy and (B) Allergic Rhinitis.
(DOC)

Figure S5 Quantile-quantile (Q-Q) plots of the observed *P* values versus the expected values from *P* value of association of the non-synonymous SNPs in the GWAS chip for (A) Atopy and (B) Allergic Rhinitis.
(DOC)

Figure S6 Quantile-quantile (Q-Q) plots of the observed *P* values versus the expected values from *P* value of association for the previously reported asthma SNPs tested for (A) Atopy and (B) Allergic Rhinitis.
(DOC)
