# Supplementary material for: Genome-Wide Association Study for Atopy and Allergic Rhinitis in a Singapore Chinese Population
Source: PLoS One. 2011 May 20;6(5):e19719. doi: 10.1371/journal.pone.0019719 (PMC3098846; doi:10.1371/journal.pone.0019719)
Supplement: Table S1 — Summary of results of SNPs selected for validation in the replication population for Atopy phenotype. (DOC) [file pone.0019719.s001.doc]

**Supplementary Table S1**: Summary of results of SNPs selected for validation in the replication population for Atopy phenotype

|  |  |  |  | **GWAS discovery phase** | | | | | | **Replication** | | | | | | **GWAS and Replication** | | | |
| --- | --- | --- | --- | --- | --- | --- | --- | --- | --- | --- | --- | --- | --- | --- | --- | --- | --- | --- | --- |
|  |  | **Minor** | **Major** | **MAF*** | |  |  | **95% CI** | | **MAF** | |  |  | **95% CI** | |  |  | **95% CI** | |
| **CHR** | **SNP** | **allele** | **allele** | **case (515)** | **control (483)** | **p-trend** | **OR** | **L95** | **U95** | **case (2323)** | **control (511)** | **p-trend** | **OR** | **L95** | **U95** | **p-trend** | **OR** | **L95** | **U95** |
| 2 | rs6716364 | T | C | 0.34 | 0.25 | 2.23E-05 | 1.54 | 1.26 | 1.88 | 0.30 | 0.28 | 1.30E-01 | 1.13 | 0.97 | 1.31 | 7.09E-05 | 1.28 | 1.13 | 1.45 |
| 19 | rs8111930 | A | G | 0.06 | 0.10 | 8.69E-05 | 0.50 | 0.35 | 0.71 | 0.09 | 0.11 | 2.98E-02 | 0.78 | 0.62 | 0.98 | 7.92E-05 | 0.69 | 0.57 | 0.83 |
| 10 | rs196342 | G | A | 0.08 | 0.04 | 7.09E-05 | 2.26 | 1.51 | 3.39 | 0.07 | 0.05 | 6.42E-02 | 1.33 | 0.98 | 1.81 | 1.15E-04 | 1.63 | 1.27 | 2.09 |
| 21 | rs2837108 | T | C | 0.05 | 0.11 | 1.05E-04 | 0.50 | 0.36 | 0.71 | 0.09 | 0.10 | 1.24E-01 | 0.84 | 0.68 | 1.05 | 2.50E-04 | 0.72 | 0.60 | 0.86 |
| 5 | rs13188584 | T | C | 0.19 | 0.13 | 1.78E-04 | 1.62 | 1.26 | 2.09 | 0.13 | 0.12 | 1.70E-01 | 1.17 | 0.93 | 1.47 | 3.29E-04 | 1.36 | 1.15 | 1.61 |
| 10 | rs505010 | C | T | 0.03 | 0.06 | 1.19E-04 | 0.40 | 0.25 | 0.64 | 0.04 | 0.05 | 6.09E-02 | 0.74 | 0.55 | 1.01 | 4.09E-04 | 0.64 | 0.50 | 0.82 |
| 6 | rs17133789 | T | G | 0.30 | 0.38 | 2.13E-04 | 0.70 | 0.57 | 0.84 | 0.33 | 0.35 | 1.16E-01 | 0.89 | 0.77 | 1.03 | 4.73E-04 | 0.82 | 0.73 | 0.92 |
| 11 | rs4573661 | G | A | 0.53 | 0.46 | 4.35E-04 | 1.38 | 1.16 | 1.66 | 0.47 | 0.50 | 8.75E-02 | 0.89 | 0.77 | 1.02 | 8.01E-04 | 0.83 | 0.74 | 0.93 |
| 7 | rs11761986 | T | G | 0.03 | 0.06 | 1.65E-04 | 0.42 | 0.27 | 0.66 | 0.04 | 0.05 | 1.63E-01 | 0.80 | 0.58 | 1.10 | 9.38E-04 | 0.66 | 0.51 | 0.84 |
| 1 | rs1001567 | G | T | 0.15 | 0.09 | 3.08E-05 | 1.86 | 1.39 | 2.49 | 0.13 | 0.12 | 3.89E-01 | 1.09 | 0.89 | 1.34 | 2.18E-03 | 1.30 | 1.10 | 1.54 |
| 20 | rs17122844 | T | C | 0.38 | 0.29 | 1.62E-04 | 1.45 | 1.20 | 1.76 | 0.33 | 0.32 | 4.34E-01 | 1.06 | 0.92 | 1.23 | 2.29E-03 | 1.20 | 1.07 | 1.35 |
| 11 | rs11230882 | A | G | 0.30 | 0.21 | 1.62E-04 | 1.49 | 1.21 | 1.84 | 0.30 | 0.29 | 7.43E-01 | 1.03 | 0.88 | 1.19 | 2.81E-03 | 1.21 | 1.07 | 1.36 |
| 20 | rs6060151 | G | T | 0.38 | 0.29 | 7.73E-05 | 1.48 | 1.22 | 1.79 | 0.33 | 0.32 | 5.41E-01 | 1.05 | 0.90 | 1.21 | 2.89E-03 | 1.20 | 1.06 | 1.34 |
| 1 | rs10493377 | A | G | 0.18 | 0.12 | 7.81E-05 | 1.70 | 1.31 | 2.21 | 0.14 | 0.13 | 3.53E-01 | 1.10 | 0.90 | 1.34 | 2.95E-03 | 1.28 | 1.09 | 1.50 |
| 3 | rs6441306 | G | A | 0.10 | 0.16 | 9.73E-05 | 0.59 | 0.45 | 0.77 | 0.12 | 0.13 | 4.10E-01 | 0.92 | 0.75 | 1.13 | 3.20E-03 | 0.79 | 0.67 | 0.92 |
| 11 | rs17822261 | T | C | 0.03 | 0.00 | 9.59E-04 | 5.98 | 2.07 | 17.29 | 0.01 | 0.01 | 6.18E-01 | 1.18 | 0.62 | 2.26 | 3.85E-03 | 2.33 | 1.31 | 4.14 |
| 2 | rs2289076 | A | G | 0.30 | 0.38 | 5.07E-05 | 0.68 | 0.56 | 0.82 | 0.33 | 0.34 | 6.38E-01 | 0.97 | 0.84 | 1.12 | 4.91E-03 | 0.85 | 0.76 | 0.95 |
| 4 | rs10034085 | G | T | 0.18 | 0.26 | 1.04E-04 | 0.65 | 0.52 | 0.81 | 0.23 | 0.24 | 7.23E-01 | 0.97 | 0.82 | 1.14 | 5.11E-03 | 0.83 | 0.73 | 0.95 |
| 12 | rs249153 | C | T | 0.13 | 0.08 | 1.18E-04 | 1.85 | 1.35 | 2.53 | 0.10 | 0.09 | 5.62E-01 | 1.07 | 0.85 | 1.35 | 5.37E-03 | 1.31 | 1.08 | 1.59 |
| 10 | rs10886849 | T | C | 0.32 | 0.41 | 2.81E-05 | 0.66 | 0.54 | 0.80 | 0.37 | 0.38 | 8.19E-01 | 0.98 | 0.85 | 1.13 | 5.83E-03 | 0.85 | 0.76 | 0.96 |
| 7 | rs7801774 | G | T | 0.43 | 0.34 | 1.09E-04 | 1.44 | 1.20 | 1.73 | 0.37 | 0.36 | 5.02E-01 | 1.05 | 0.91 | 1.21 | 5.88E-03 | 1.17 | 1.05 | 1.31 |
| 12 | rs1001484 | A | G | 0.23 | 0.32 | 4.47E-04 | 0.70 | 0.57 | 0.85 | 0.26 | 0.26 | 8.84E-01 | 0.99 | 0.84 | 1.16 | 5.98E-03 | 0.84 | 0.75 | 0.95 |
| 21 | rs2826877 | C | T | 0.38 | 0.46 | 1.54E-04 | 0.70 | 0.58 | 0.84 | 0.40 | 0.41 | 6.39E-01 | 0.97 | 0.84 | 1.11 | 7.34E-03 | 0.86 | 0.77 | 0.96 |
| 12 | rs12312641 | A | G | 0.05 | 0.10 | 1.21E-04 | 0.50 | 0.35 | 0.71 | 0.08 | 0.09 | 6.53E-01 | 0.95 | 0.74 | 1.21 | 9.47E-03 | 0.77 | 0.64 | 0.94 |
| 4 | rs1480990 | T | C | 0.03 | 0.06 | 9.04E-05 | 0.38 | 0.23 | 0.61 | 0.04 | 0.04 | 9.57E-01 | 0.99 | 0.69 | 1.42 | 1.32E-02 | 0.72 | 0.55 | 0.93 |
| 10 | rs11199746 | G | A | 0.21 | 0.29 | 2.40E-05 | 0.64 | 0.51 | 0.78 | 0.26 | 0.26 | 8.67E-01 | 0.99 | 0.84 | 1.15 | 1.41E-02 | 0.86 | 0.76 | 0.97 |
| 7 | rs4074751 | G | T | 0.32 | 0.25 | 3.20E-04 | 1.45 | 1.18 | 1.77 | 0.31 | 0.30 | 4.91E-01 | 1.05 | 0.91 | 1.22 | 1.53E-02 | 1.16 | 1.03 | 1.31 |
| 1 | rs759914 | T | G | 0.32 | 0.25 | 6.16E-05 | 1.52 | 1.24 | 1.86 | 0.29 | 0.29 | 8.65E-01 | 1.01 | 0.87 | 1.18 | 2.04E-02 | 1.16 | 1.02 | 1.31 |
| 21 | rs2822793 | T | C | 0.05 | 0.09 | 3.73E-04 | 0.50 | 0.35 | 0.74 | 0.07 | 0.07 | 8.77E-01 | 0.98 | 0.75 | 1.29 | 2.28E-02 | 0.78 | 0.64 | 0.97 |
| 18 | rs878396 | A | G | 0.21 | 0.29 | 3.85E-05 | 0.64 | 0.52 | 0.79 | 0.25 | 0.25 | 7.28E-01 | 1.03 | 0.88 | 1.21 | 3.20E-02 | 0.87 | 0.77 | 0.99 |
| 1 | rs11260978 | A | G | 0.24 | 0.17 | 3.91E-04 | 1.51 | 1.20 | 1.90 | 0.21 | 0.21 | 6.61E-01 | 0.96 | 0.81 | 1.14 | 4.16E-02 | 1.15 | 1.01 | 1.32 |
| 5 | rs2112197 | C | T | 0.30 | 0.23 | 2.85E-05 | 1.56 | 1.27 | 1.92 | 0.24 | 0.25 | 6.83E-01 | 0.97 | 0.83 | 1.13 | 4.75E-02 | 1.14 | 1.00 | 1.29 |
| 3 | rs6443904 | G | A | 0.35 | 0.29 | 4.91E-04 | 1.42 | 1.16 | 1.72 | 0.33 | 0.32 | 8.14E-01 | 1.02 | 0.88 | 1.18 | 4.99E-02 | 1.13 | 1.00 | 1.27 |
| 5 | rs626105 | A | G | 0.23 | 0.17 | 1.20E-04 | 1.55 | 1.24 | 1.95 | 0.19 | 0.20 | 5.68E-01 | 0.95 | 0.80 | 1.13 | 6.24E-02 | 1.14 | 0.99 | 1.31 |
| 9 | rs13284515 | T | C | 0.14 | 0.09 | 3.36E-04 | 1.70 | 1.27 | 2.27 | 0.12 | 0.12 | 6.19E-01 | 0.95 | 0.77 | 1.17 | 8.51E-02 | 1.16 | 0.98 | 1.38 |
| 6 | rs17085260 | C | A | 0.46 | 0.38 | 2.27E-04 | 1.41 | 1.17 | 1.69 | 0.43 | 0.44 | 5.60E-01 | 0.96 | 0.84 | 1.10 | 9.46E-02 | 1.10 | 0.98 | 1.22 |
| 9 | rs7020934 | T | C | 0.11 | 0.08 | 4.64E-04 | 1.76 | 1.28 | 2.42 | 0.12 | 0.12 | 9.50E-01 | 0.99 | 0.80 | 1.23 | 9.65E-02 | 1.16 | 0.97 | 1.39 |
| 7 | rs10278663 | A | G | 0.30 | 0.38 | 7.07E-05 | 0.68 | 0.56 | 0.82 | 0.33 | 0.32 | 2.95E-01 | 1.08 | 0.93 | 1.25 | 1.39E-01 | 0.92 | 0.82 | 1.03 |
| 18 | rs2852950 | G | A | 0.37 | 0.28 | 1.86E-05 | 1.52 | 1.26 | 1.85 | 0.31 | 0.33 | 1.10E-01 | 0.89 | 0.77 | 1.03 | 1.52E-01 | 1.09 | 0.97 | 1.22 |
| 14 | rs210360 | C | T | 0.37 | 0.46 | 6.12E-05 | 0.69 | 0.57 | 0.83 | 0.41 | 0.39 | 1.75E-01 | 1.10 | 0.96 | 1.27 | 1.85E-01 | 0.93 | 0.83 | 1.04 |
| 8 | rs2738133 | A | G | 0.33 | 0.27 | 1.95E-04 | 1.47 | 1.20 | 1.80 | 0.29 | 0.30 | 3.59E-01 | 0.93 | 0.80 | 1.08 | 2.33E-01 | 1.08 | 0.95 | 1.21 |
| 18 | rs2044107 | A | G | 0.03 | 0.01 | 5.24E-04 | 3.64 | 1.75 | 7.56 | 0.03 | 0.03 | 1.40E-01 | 0.75 | 0.51 | 1.10 | 3.35E-01 | 1.19 | 0.83 | 1.70 |
| 5 | rs17702421 | C | A | 0.30 | 0.37 | 7.09E-05 | 0.68 | 0.56 | 0.82 | 0.35 | 0.33 | 7.49E-02 | 1.14 | 0.99 | 1.32 | 3.79E-01 | 0.95 | 0.85 | 1.06 |
| 4 | rs897945 | T | G | 0.19 | 0.16 | 2.64E-03 | 1.45 | 1.14 | 1.86 | 0.18 | 0.21 | 2.89E-02 | 0.83 | 0.70 | 0.98 | 6.75E-01 | 0.97 | 0.84 | 1.12 |
| 2 | rs1350342 | T | C | 0.02 | 0.05 | 3.09E-04 | 0.38 | 0.22 | 0.64 | NA | NA | NA | NA | NA | NA | NA | NA | NA | NA |
| 7 | rs4727753 | G | A | 0.22 | 0.16 | 8.64E-05 | 1.60 | 1.27 | 2.02 | 0.19 | 0.16 | 8.75E-02 | 1.17 | 0.98 | 1.41 | 7.04E-04 | 1.28 | 1.11 | 1.48 |
| 7 | rs6948090 | A | G | 0.24 | 0.16 | 2.87E-06 | 1.74 | 1.38 | 2.20 | 0.20 | 0.19 | 4.41E-01 | 1.07 | 0.90 | 1.27 | 8.47E-04 | 1.27 | 1.10 | 1.45 |
| 20 | rs17122844 | A | G | 0.38 | 0.29 | 1.62E-04 | 1.45 | 1.20 | 1.76 | 0.33 | 0.32 | 4.28E-01 | 1.06 | 0.92 | 1.23 | 2.54E-03 | 1.20 | 1.07 | 1.35 |
| 20 | rs2145557 | A | G | 0.37 | 0.29 | 8.13E-05 | 1.48 | 1.22 | 1.79 | 0.33 | 0.32 | 5.60E-01 | 1.05 | 0.90 | 1.21 | 3.21E-03 | 1.19 | 1.06 | 1.34 |
| 8 | rs10505427 | A | G | 0.04 | 0.09 | 5.95E-05 | 0.46 | 0.32 | 0.68 | 0.06 | 0.06 | 7.48E-01 | 0.95 | 0.72 | 1.27 | 5.69E-03 | 0.74 | 0.60 | 0.92 |
| 1 | rs12133327 | G | A | 0.21 | 0.29 | 5.63E-05 | 0.65 | 0.53 | 0.80 | 0.23 | 0.24 | 7.71E-01 | 0.98 | 0.83 | 1.15 | 5.94E-03 | 0.84 | 0.74 | 0.95 |
| 3 | rs9310496 | G | A | 0.22 | 0.15 | 1.02E-04 | 1.58 | 1.25 | 1.98 | 0.19 | 0.19 | 7.57E-01 | 1.03 | 0.86 | 1.23 | 6.33E-03 | 1.22 | 1.06 | 1.40 |
| 7 | rs273957 | G | A | 0.05 | 0.09 | 6.69E-04 | 0.52 | 0.36 | 0.76 | 0.08 | 0.08 | 4.77E-01 | 0.91 | 0.72 | 1.17 | 9.09E-03 | 0.77 | 0.63 | 0.94 |
| 1 | rs1041238 | G | A | 0.21 | 0.29 | 6.08E-05 | 0.65 | 0.53 | 0.80 | 0.23 | 0.23 | 9.37E-01 | 0.99 | 0.85 | 1.17 | 1.02E-02 | 0.85 | 0.75 | 0.96 |
| 18 | rs8085335 | G | A | 0.21 | 0.14 | 1.81E-04 | 1.57 | 1.24 | 1.98 | 0.17 | 0.17 | 9.60E-01 | 1.01 | 0.84 | 1.21 | 1.65E-02 | 1.20 | 1.03 | 1.38 |
| 1 | rs11260978 | A | G | 0.24 | 0.17 | 3.91E-04 | 1.51 | 1.20 | 1.90 | 0.21 | 0.21 | 8.51E-01 | 0.98 | 0.83 | 1.17 | 2.12E-02 | 1.18 | 1.03 | 1.35 |
| 7 | rs4722378 | G | A | 0.45 | 0.54 | 7.67E-05 | 0.69 | 0.58 | 0.83 | 0.49 | 0.48 | 6.95E-01 | 1.03 | 0.90 | 1.18 | 3.45E-02 | 0.89 | 0.80 | 0.99 |
| 7 | rs2237315 | C | A | 0.46 | 0.54 | 9.09E-05 | 0.70 | 0.58 | 0.84 | 0.49 | 0.49 | 7.88E-01 | 1.02 | 0.89 | 1.17 | 3.57E-02 | 0.89 | 0.80 | 0.99 |
| 7 | rs2237310 | C | A | 0.44 | 0.53 | 1.11E-04 | 0.70 | 0.59 | 0.84 | 0.47 | 0.47 | 6.80E-01 | 1.03 | 0.90 | 1.18 | 4.34E-02 | 0.89 | 0.80 | 1.00 |
| 7 | rs799623 | G | A | 0.19 | 0.13 | 6.71E-05 | 1.68 | 1.30 | 2.16 | 0.16 | 0.16 | 7.58E-01 | 0.97 | 0.81 | 1.17 | 5.32E-02 | 1.16 | 1.00 | 1.35 |
| 7 | rs10267134 | G | A | 0.30 | 0.38 | 7.07E-05 | 0.68 | 0.56 | 0.82 | 0.33 | 0.32 | 5.20E-01 | 1.05 | 0.91 | 1.22 | 6.64E-02 | 0.90 | 0.80 | 1.01 |
| 7 | rs12700538 | A | G | 0.45 | 0.54 | 6.00E-05 | 0.69 | 0.57 | 0.83 | 0.48 | 0.47 | 3.62E-01 | 1.07 | 0.93 | 1.23 | 7.98E-02 | 0.91 | 0.81 | 1.01 |
| 6 | rs17085260 | C | A | 0.46 | 0.38 | 2.27E-04 | 1.41 | 1.17 | 1.69 | 0.43 | 0.44 | 5.50E-01 | 0.96 | 0.83 | 1.10 | 8.70E-02 | 1.10 | 0.99 | 1.23 |
| 19 | rs9305012 | G | A | 0.51 | 0.43 | 8.52E-04 | 1.37 | 1.14 | 1.64 | 0.45 | 0.47 | 3.56E-01 | 0.94 | 0.81 | 1.08 | 1.52E-01 | 1.08 | 0.97 | 1.21 |
| 8 | rs2472553 | A | G | 0.45 | 0.38 | 6.22E-04 | 1.37 | 1.15 | 1.65 | 0.43 | 0.44 | 4.32E-01 | 0.95 | 0.82 | 1.09 | 1.57E-01 | 1.08 | 0.97 | 1.21 |
| 7 | rs324389 | A | G | 0.42 | 0.51 | 5.77E-05 | 0.69 | 0.57 | 0.83 | 0.44 | 0.42 | 1.39E-01 | 1.11 | 0.97 | 1.28 | 1.91E-01 | 0.93 | 0.83 | 1.04 |
| 19 | rs1058402 | A | G | 0.13 | 0.09 | 7.38E-04 | 1.67 | 1.24 | 2.24 | 0.10 | 0.11 | 2.25E-01 | 0.87 | 0.70 | 1.09 | 2.28E-01 | 1.12 | 0.93 | 1.34 |
| 7 | rs10270663 | A | C | 0.41 | 0.51 | 3.56E-05 | 0.68 | 0.57 | 0.82 | 0.44 | 0.41 | 8.52E-02 | 1.13 | 0.98 | 1.30 | 2.30E-01 | 0.94 | 0.84 | 1.04 |

***** *MAF - Minor allele frequency*
