# Supplementary material for: Genome-Wide Association Study for Atopy and Allergic Rhinitis in a Singapore Chinese Population
Source: PLoS One. 2011 May 20;6(5):e19719. doi: 10.1371/journal.pone.0019719 (PMC3098846; doi:10.1371/journal.pone.0019719)
Supplement: Table S2 — Summary of results of SNPs selected for validation in the replication population for AR phenotype. (DOC) [file pone.0019719.s002.doc]

**Supplementary Table S2: Summary of results of SNPs selected for validation in the replication population for AR phenotype**

|  |  |  |  | **GWAS** | | | | | | **Replication** | | | | | | **GWAS and Replication**  **95% CI** | | | |
| --- | --- | --- | --- | --- | --- | --- | --- | --- | --- | --- | --- | --- | --- | --- | --- | --- | --- | --- | --- |
|  |  | **Minor**  **allele** | **Major**  **allele** | **MAF*** | |  |  | **95% CI** | | **MAF*** | |  |  | **95% CI** | |  |  |  |  |
| **CHR** | **SNP** |  |  | **case (456)** | **control (483)** | **p-trend** | **OR** | **L95** | **U95** | **Case (676)** | **Control (511)** | **p-trend** | **OR** | **L95** | **U95** | **p-trend** | **OR** | **L95** | **U95** |
| 19 | rs8111930 | A | G | 0.05 | 0.10 | 1.25E-04 | 0.49 | 0.34 | 0.71 | 0.08 | 0.11 | 4.95E-02 | 0.76 | 0.57 | 1.00 | 6.61E-05 | 0.64 | 0.51 | 0.80 |
| 5 | rs13188584 | T | C | 0.19 | 0.13 | 2.29E-04 | 1.62 | 1.25 | 2.10 | 0.14 | 0.12 | 7.77E-02 | 1.27 | 0.97 | 1.66 | 7.56E-05 | 1.46 | 1.21 | 1.75 |
| 2 | rs6716364 | T | C | 0.34 | 0.25 | 6.70E-06 | 1.61 | 1.31 | 1.98 | 0.31 | 0.28 | 8.34E-02 | 1.17 | 0.98 | 1.41 | 1.01E-04 | 1.32 | 1.15 | 1.51 |
| 6 | rs17133789 | T | G | 0.29 | 0.38 | 1.15E-05 | 0.64 | 0.53 | 0.78 | 0.33 | 0.35 | 1.68E-01 | 0.89 | 0.75 | 1.05 | 1.04E-04 | 0.77 | 0.68 | 0.88 |
| 10 | rs505010 | C | T | 0.03 | 0.06 | 1.11E-03 | 0.44 | 0.27 | 0.72 | 0.03 | 0.05 | 2.69E-02 | 0.63 | 0.42 | 0.95 | 1.34E-04 | 0.54 | 0.39 | 0.74 |
| 11 | rs11230882 | A | G | 0.31 | 0.21 | 1.32E-06 | 1.70 | 1.37 | 2.10 | 0.31 | 0.29 | 5.05E-01 | 1.06 | 0.89 | 1.28 | 2.34E-04 | 1.30 | 1.13 | 1.50 |
| 10 | rs196342 | G | A | 0.09 | 0.04 | 1.14E-04 | 2.23 | 1.48 | 3.35 | 0.06 | 0.05 | 3.39E-01 | 1.19 | 0.83 | 1.72 | 5.84E-04 | 1.62 | 1.23 | 2.12 |
| 11 | rs4573661 | G | A | 0.54 | 0.46 | 1.56E-03 | 1.35 | 1.12 | 1.62 | 0.47 | 0.50 | 1.23E-01 | 0.88 | 0.74 | 1.04 | 6.24E-04 | 0.80 | 0.71 | 0.91 |
| 7 | rs7801774 | G | T | 0.44 | 0.34 | 2.79E-05 | 1.49 | 1.24 | 1.80 | 0.37 | 0.36 | 3.84E-01 | 1.08 | 0.91 | 1.28 | 6.65E-04 | 1.25 | 1.10 | 1.42 |
| 1 | rs10493377 | A | G | 0.17 | 0.12 | 1.52E-03 | 1.54 | 1.18 | 2.01 | 0.16 | 0.13 | 9.52E-02 | 1.22 | 0.97 | 1.55 | 9.50E-04 | 1.35 | 1.13 | 1.62 |
| 21 | rs2837108 | T | C | 0.05 | 0.10 | 1.18E-04 | 0.49 | 0.34 | 0.70 | 0.09 | 0.10 | 3.23E-01 | 0.88 | 0.68 | 1.14 | 1.29E-03 | 0.71 | 0.57 | 0.87 |
| 7 | rs11761986 | T | G | 0.03 | 0.07 | 2.22E-04 | 0.41 | 0.25 | 0.66 | 0.04 | 0.05 | 2.58E-01 | 0.80 | 0.54 | 1.18 | 1.45E-03 | 0.61 | 0.46 | 0.83 |
| 3 | rs6441306 | G | A | 0.11 | 0.16 | 3.23E-04 | 0.61 | 0.46 | 0.80 | 0.12 | 0.13 | 3.56E-01 | 0.89 | 0.70 | 1.14 | 1.48E-03 | 0.74 | 0.62 | 0.89 |
| 11 | rs17822261 | T | C | 0.03 | 0.00 | 2.39E-04 | 7.41 | 2.55 | 21.58 | 0.01 | 0.01 | 9.13E-01 | 0.96 | 0.43 | 2.13 | 2.71E-03 | 2.53 | 1.38 | 4.64 |
| 12 | rs249153 | C | T | 0.13 | 0.08 | 3.94E-04 | 1.79 | 1.30 | 2.47 | 0.10 | 0.09 | 3.91E-01 | 1.13 | 0.86 | 1.49 | 2.95E-03 | 1.38 | 1.12 | 1.71 |
| 7 | rs4074751 | G | T | 0.33 | 0.25 | 2.18E-04 | 1.47 | 1.20 | 1.81 | 0.31 | 0.30 | 4.14E-01 | 1.08 | 0.90 | 1.28 | 4.72E-03 | 1.21 | 1.06 | 1.39 |
| 12 | rs1001484 | A | G | 0.23 | 0.32 | 8.91E-05 | 0.66 | 0.54 | 0.81 | 0.26 | 0.26 | 9.06E-01 | 0.99 | 0.82 | 1.19 | 7.04E-03 | 0.83 | 0.72 | 0.95 |
| 4 | rs10034085 | G | T | 0.19 | 0.26 | 1.41E-04 | 0.65 | 0.52 | 0.81 | 0.23 | 0.24 | 8.57E-01 | 0.98 | 0.81 | 1.19 | 7.42E-03 | 0.82 | 0.71 | 0.95 |
| 18 | rs878396 | A | G | 0.21 | 0.29 | 9.74E-05 | 0.65 | 0.52 | 0.81 | 0.25 | 0.25 | 9.85E-01 | 1.00 | 0.83 | 1.21 | 8.42E-03 | 0.83 | 0.72 | 0.95 |
| 3 | rs6443904 | G | A | 0.36 | 0.29 | 1.21E-03 | 1.39 | 1.14 | 1.69 | 0.33 | 0.32 | 6.69E-01 | 1.04 | 0.87 | 1.24 | 8.49E-03 | 1.20 | 1.05 | 1.37 |
| 21 | rs2826877 | C | T | 0.37 | 0.46 | 2.00E-04 | 0.69 | 0.57 | 0.84 | 0.40 | 0.41 | 8.64E-01 | 0.99 | 0.83 | 1.17 | 9.39E-03 | 0.84 | 0.74 | 0.96 |
| 4 | rs1480990 | T | C | 0.03 | 0.06 | 8.35E-04 | 0.43 | 0.26 | 0.70 | 0.03 | 0.04 | 5.34E-01 | 0.87 | 0.55 | 1.36 | 1.05E-02 | 0.65 | 0.47 | 0.91 |
| 12 | rs12312641 | A | G | 0.06 | 0.10 | 5.17E-04 | 0.52 | 0.36 | 0.75 | 0.08 | 0.09 | 7.41E-01 | 0.95 | 0.71 | 1.28 | 1.07E-02 | 0.74 | 0.59 | 0.93 |
| 20 | rs17122844 | T | C | 0.38 | 0.29 | 2.02E-04 | 1.45 | 1.19 | 1.77 | 0.32 | 0.32 | 9.26E-01 | 0.99 | 0.83 | 1.18 | 1.20E-02 | 1.18 | 1.04 | 1.35 |
| 1 | rs11260978 | A | G | 0.25 | 0.17 | 1.27E-05 | 1.68 | 1.33 | 2.13 | 0.21 | 0.21 | 7.05E-01 | 0.96 | 0.78 | 1.18 | 1.23E-02 | 1.22 | 1.04 | 1.42 |
| 20 | rs6060151 | G | T | 0.38 | 0.29 | 8.68E-05 | 1.49 | 1.22 | 1.81 | 0.32 | 0.32 | 8.37E-01 | 0.98 | 0.83 | 1.17 | 1.41E-02 | 1.18 | 1.03 | 1.34 |
| 10 | rs10886849 | T | C | 0.34 | 0.42 | 4.19E-04 | 0.70 | 0.57 | 0.85 | 0.37 | 0.38 | 6.92E-01 | 0.97 | 0.82 | 1.15 | 1.44E-02 | 0.85 | 0.75 | 0.97 |
| 1 | rs1001567 | G | T | 0.15 | 0.09 | 2.18E-04 | 1.75 | 1.30 | 2.35 | 0.13 | 0.12 | 8.68E-01 | 1.02 | 0.80 | 1.30 | 1.45E-02 | 1.27 | 1.05 | 1.53 |
| 2 | rs2289076 | A | G | 0.30 | 0.38 | 1.96E-04 | 0.69 | 0.57 | 0.84 | 0.34 | 0.34 | 9.00E-01 | 1.01 | 0.85 | 1.20 | 1.93E-02 | 0.86 | 0.75 | 0.98 |
| 21 | rs2822793 | T | C | 0.04 | 0.08 | 5.60E-04 | 0.50 | 0.34 | 0.74 | 0.07 | 0.07 | 9.22E-01 | 1.02 | 0.73 | 1.41 | 2.22E-02 | 0.75 | 0.58 | 0.96 |
| 5 | rs626105 | A | G | 0.24 | 0.17 | 5.52E-04 | 1.50 | 1.19 | 1.88 | 0.19 | 0.20 | 5.84E-01 | 0.94 | 0.77 | 1.16 | 2.44E-02 | 1.20 | 1.02 | 1.40 |
| 6 | rs17085260 | C | A | 0.46 | 0.38 | 7.54E-04 | 1.38 | 1.14 | 1.66 | 0.44 | 0.44 | 9.33E-01 | 1.01 | 0.86 | 1.19 | 2.88E-02 | 1.15 | 1.01 | 1.30 |
| 8 | rs2738133 | A | G | 0.34 | 0.27 | 9.99E-04 | 1.41 | 1.15 | 1.74 | 0.30 | 0.30 | 8.79E-01 | 0.99 | 0.83 | 1.18 | 3.23E-02 | 1.16 | 1.01 | 1.33 |
| 10 | rs11199746 | G | A | 0.22 | 0.29 | 3.37E-03 | 0.73 | 0.59 | 0.90 | 0.25 | 0.26 | 7.73E-01 | 0.97 | 0.81 | 1.17 | 4.08E-02 | 0.86 | 0.75 | 0.99 |
| 18 | rs2852950 | G | A | 0.37 | 0.28 | 8.31E-05 | 1.49 | 1.22 | 1.81 | 0.32 | 0.33 | 3.28E-01 | 0.92 | 0.77 | 1.09 | 6.06E-02 | 1.14 | 0.99 | 1.30 |
| 9 | rs7020934 | T | C | 0.12 | 0.07 | 3.83E-03 | 1.61 | 1.17 | 2.22 | 0.11 | 0.12 | 7.95E-01 | 0.97 | 0.75 | 1.25 | 7.95E-02 | 1.20 | 0.98 | 1.46 |
| 14 | rs210360 | C | T | 0.36 | 0.46 | 2.49E-05 | 0.67 | 0.55 | 0.81 | 0.42 | 0.39 | 1.60E-01 | 1.13 | 0.95 | 1.34 | 8.05E-02 | 0.89 | 0.79 | 1.01 |
| 1 | rs759914 | T | G | 0.33 | 0.25 | 7.46E-04 | 1.43 | 1.16 | 1.75 | 0.28 | 0.29 | 6.08E-01 | 0.95 | 0.80 | 1.14 | 8.89E-02 | 1.13 | 0.98 | 1.29 |
| 5 | rs17702421 | C | A | 0.30 | 0.37 | 1.77E-03 | 0.74 | 0.61 | 0.89 | 0.35 | 0.33 | 3.09E-01 | 1.10 | 0.92 | 1.31 | 9.44E-02 | 0.89 | 0.78 | 1.02 |
| 9 | rs13284515 | T | C | 0.14 | 0.09 | 5.31E-04 | 1.67 | 1.25 | 2.24 | 0.10 | 0.12 | 1.12E-01 | 0.82 | 0.63 | 1.05 | 2.39E-01 | 1.12 | 0.93 | 1.36 |
| 5 | rs2112197 | C | T | 0.30 | 0.23 | 1.97E-03 | 1.39 | 1.13 | 1.72 | 0.22 | 0.25 | 6.92E-02 | 0.84 | 0.69 | 1.01 | 3.91E-01 | 1.06 | 0.92 | 1.23 |
| 4 | rs897945 | T | G | 0.20 | 0.15 | 1.08E-02 | 1.38 | 1.08 | 1.76 | 0.19 | 0.21 | 1.51E-01 | 0.86 | 0.70 | 1.06 | 4.54E-01 | 1.06 | 0.91 | 1.25 |
| 7 | rs10278663 | A | G | 0.30 | 0.38 | 4.36E-04 | 0.70 | 0.58 | 0.85 | 0.36 | 0.32 | 3.19E-02 | 1.21 | 1.02 | 1.44 | 5.37E-01 | 0.96 | 0.84 | 1.09 |
| 18 | rs2044107 | A | G | 0.04 | 0.01 | 1.71E-04 | 4.08 | 1.96 | 8.50 | 0.02 | 0.03 | 4.60E-03 | 0.45 | 0.26 | 0.78 | 5.83E-01 | 1.12 | 0.74 | 1.70 |
| 2 | rs1350342 | T | C | 0.02 | 0.05 | 3.10E-04 | 0.35 | 0.20 | 0.62 | NA | NA | NA | NA | NA | NA | NA | NA | NA | NA |
| 7 | rs4727753 | G | A | 0.22 | 0.16 | 6.58E-05 | 1.65 | 1.29 | 2.11 | 0.19 | 0.16 | 6.98E-02 | 1.22 | 0.98 | 1.52 | 2.17E-04 | 1.35 | 1.15 | 1.59 |
| 7 | rs6948090 | A | G | 0.24 | 0.16 | 2.47E-06 | 1.79 | 1.41 | 2.28 | 0.20 | 0.19 | 6.45E-01 | 1.05 | 0.85 | 1.30 | 6.18E-04 | 1.32 | 1.12 | 1.54 |
| 8 | rs10505427 | A | G | 0.04 | 0.09 | 5.86E-05 | 0.45 | 0.30 | 0.66 | 0.05 | 0.06 | 4.12E-01 | 0.86 | 0.60 | 1.23 | 8.80E-04 | 0.65 | 0.50 | 0.84 |
| 1 | rs12133327 | G | A | 0.21 | 0.29 | 1.07E-04 | 0.65 | 0.53 | 0.81 | 0.23 | 0.24 | 5.16E-01 | 0.94 | 0.77 | 1.14 | 1.21E-03 | 0.79 | 0.69 | 0.91 |
| 1 | rs1041238 | G | A | 0.21 | 0.29 | 1.16E-04 | 0.65 | 0.53 | 0.81 | 0.22 | 0.23 | 5.61E-01 | 0.94 | 0.78 | 1.15 | 1.50E-03 | 0.80 | 0.69 | 0.92 |
| 18 | rs8085335 | G | A | 0.21 | 0.14 | 3.52E-04 | 1.56 | 1.22 | 1.99 | 0.18 | 0.17 | 4.41E-01 | 1.09 | 0.88 | 1.36 | 1.86E-03 | 1.29 | 1.10 | 1.52 |
| 3 | rs9310496 | G | A | 0.22 | 0.15 | 2.10E-04 | 1.57 | 1.24 | 1.99 | 0.20 | 0.19 | 4.73E-01 | 1.08 | 0.88 | 1.32 | 2.40E-03 | 1.27 | 1.09 | 1.48 |
| 1 | rs11260978 | A | G | 0.25 | 0.17 | 5.01E-05 | 1.64 | 1.29 | 2.08 | 0.20 | 0.21 | 7.98E-01 | 0.97 | 0.79 | 1.20 | 5.46E-03 | 1.25 | 1.07 | 1.45 |
| 7 | rs273957 | G | A | 0.05 | 0.09 | 3.55E-03 | 0.56 | 0.38 | 0.83 | 0.08 | 0.08 | 4.65E-01 | 0.89 | 0.66 | 1.21 | 1.10E-02 | 0.74 | 0.58 | 0.93 |
| 20 | rs17122844 | A | G | 0.38 | 0.29 | 4.40E-05 | 1.52 | 1.25 | 1.87 | 0.32 | 0.32 | 9.80E-01 | 1.00 | 0.84 | 1.19 | 1.19E-02 | 1.18 | 1.04 | 1.35 |
| 20 | rs2145557 | A | G | 0.38 | 0.29 | 1.92E-05 | 1.56 | 1.27 | 1.91 | 0.32 | 0.32 | 8.28E-01 | 0.98 | 0.82 | 1.17 | 1.33E-02 | 1.18 | 1.04 | 1.34 |
| 6 | rs17085260 | C | A | 0.46 | 0.38 | 5.26E-05 | 1.49 | 1.23 | 1.80 | 0.44 | 0.44 | 9.09E-01 | 1.01 | 0.86 | 1.19 | 2.01E-02 | 1.16 | 1.02 | 1.31 |
| 7 | rs799623 | G | A | 0.19 | 0.13 | 7.10E-05 | 1.72 | 1.32 | 2.25 | 0.15 | 0.16 | 5.46E-01 | 0.93 | 0.75 | 1.17 | 4.54E-02 | 1.19 | 1.00 | 1.41 |
| 19 | rs9305012 | G | A | 0.51 | 0.43 | 1.66E-03 | 1.36 | 1.12 | 1.65 | 0.45 | 0.47 | 3.63E-01 | 0.93 | 0.78 | 1.09 | 7.17E-02 | 1.12 | 0.99 | 1.27 |
| 7 | rs2237315 | A | C | 0.54 | 0.46 | 6.28E-04 | 1.38 | 1.15 | 1.67 | 0.50 | 0.49 | 4.72E-01 | 1.06 | 0.90 | 1.25 | 9.21E-02 | 0.90 | 0.80 | 1.02 |
| 19 | rs1058402 | A | G | 0.13 | 0.09 | 7.54E-04 | 1.69 | 1.25 | 2.30 | 0.10 | 0.11 | 3.54E-01 | 0.88 | 0.67 | 1.15 | 1.00E-01 | 1.18 | 0.97 | 1.44 |
| 7 | rs4722378 | G | A | 0.45 | 0.54 | 5.13E-04 | 0.72 | 0.59 | 0.86 | 0.50 | 0.48 | 3.07E-01 | 1.09 | 0.92 | 1.29 | 1.02E-01 | 0.90 | 0.80 | 1.02 |
| 7 | rs2237310 | C | A | 0.44 | 0.53 | 5.17E-04 | 0.72 | 0.60 | 0.87 | 0.49 | 0.47 | 3.13E-01 | 1.09 | 0.92 | 1.29 | 1.09E-01 | 0.90 | 0.80 | 1.02 |
| 7 | rs12700538 | A | G | 0.45 | 0.54 | 4.27E-04 | 0.71 | 0.59 | 0.86 | 0.50 | 0.47 | 1.40E-01 | 1.14 | 0.96 | 1.35 | 1.77E-01 | 0.92 | 0.81 | 1.04 |
| 8 | rs2472553 | A | G | 0.45 | 0.38 | 1.59E-03 | 1.36 | 1.12 | 1.65 | 0.42 | 0.44 | 2.30E-01 | 0.90 | 0.77 | 1.07 | 2.77E-01 | 1.07 | 0.95 | 1.21 |
| 7 | rs10267134 | G | A | 0.30 | 0.38 | 1.03E-04 | 0.67 | 0.55 | 0.82 | 0.36 | 0.32 | 5.97E-02 | 1.18 | 0.99 | 1.41 | 3.63E-01 | 0.94 | 0.83 | 1.07 |
| 7 | rs324389 | A | G | 0.42 | 0.51 | 1.45E-04 | 0.69 | 0.57 | 0.84 | 0.47 | 0.42 | 1.50E-02 | 1.23 | 1.04 | 1.46 | 4.64E-01 | 0.95 | 0.84 | 1.08 |
| 7 | rs10270663 | A | C | 0.42 | 0.51 | 9.96E-05 | 0.69 | 0.57 | 0.83 | 0.47 | 0.41 | 7.05E-03 | 1.26 | 1.07 | 1.49 | 5.44E-01 | 0.96 | 0.85 | 1.09 |

******* *MAF – Minor Allele Frequency*
