# Supplementary material for: Genome-Wide Association Study for Atopy and Allergic Rhinitis in a Singapore Chinese Population
Source: PLoS One. 2011 May 20;6(5):e19719. doi: 10.1371/journal.pone.0019719 (PMC3098846; doi:10.1371/journal.pone.0019719)
Supplement: Table S3 — Predicted effect of SNPs validated in replication study on Transcription Factor Binding Sites. (DOC) [file pone.0019719.s003.doc]

**Supplementary Table S3:** Predicted effect of SNPs validated in replication study on Transcription Factor Binding Sites

| **SNPid (rs)** | **Position** | **Alleles** | **Effect** | **Family/matrix** | **Optimum**  **threshold** | **Start**  **position** | **End position** | **Strand** | **Core**  **similarity score** | **Matrix**  **similarity score** |
| --- | --- | --- | --- | --- | --- | --- | --- | --- | --- | --- |
| rs505010 | 201 | C -> T | lost | V$NFAT/NFAT.01 | 0.95 | 189 | 207 | - | 1 | 0.96 |
| rs505010 | 201 | C -> T | lost | V$ETSF/PU1.01 | 0.89 | 190 | 210 | - | 1 | 0.894 |
| rs505010 | 201 | C -> T | new | V$E2FF/E2F.01 | 0.75 | 195 | 211 | - | 1 | 0.839 |
| rs8111930 | 208 | A -> G | lost | V$ZFHX/AREB6.02 | 0.97 | 206 | 218 | + | 1 | 0.97 |
| rs8111930 | 208 | A -> G | new | V$CREB/CREB.02 | 0.89 | 200 | 220 | - | 1 | 0.918 |
