# Supplementary material for: Genome-Wide Association Study for Atopy and Allergic Rhinitis in a Singapore Chinese Population
Source: PLoS One. 2011 May 20;6(5):e19719. doi: 10.1371/journal.pone.0019719 (PMC3098846; doi:10.1371/journal.pone.0019719)
Supplement: Table S4 — Further information on Transcription Factor Binding Sites (TFBS). (DOC) [file pone.0019719.s004.doc]

**Supplementary Table S4: Further information on Transcription Factor Binding Sites (TFBS)**

| **Family/matrix** | **Further information** |
| --- | --- |
| V$NFAT/NFAT.01 | Nuclear factor of activated T-cells |
| V$ETSF/PU1.01 | Pu.1 (Pu120) Ets-like transcription factor identified in lymphoid B-cells |
| V$E2FF/E2F.01 | E2F, involved in cell cycle regulation, interacts with Rb p107 protein |
| V$ZFHX/AREB6.02 | AREB6 (Atp1a1 regulatory element binding factor 6) |
| V$CREB/CREB.02 | cAMP-responsive element binding protein |
