# Supplementary material for: Genome-Wide Association Study for Atopy and Allergic Rhinitis in a Singapore Chinese Population
Source: PLoS One. 2011 May 20;6(5):e19719. doi: 10.1371/journal.pone.0019719 (PMC3098846; doi:10.1371/journal.pone.0019719)
Supplement: Table S5 — P-values of SNPs from previous GWAS reported loci for asthma phenotype in Singapore Chinese GWAS. (DOC) [file pone.0019719.s005.doc]

**Supplementary Table S5: P-values of SNPs from previous GWAS reported loci for asthma phenotype in Singapore Chinese GWAS**

| **SNP id (rs)** | **Alleles** | **Strand** | **Chr** | **Coordinate** | **Gene Symbol** | **Position** | **Atopy p-value** | **AR p-value** |
| --- | --- | --- | --- | --- | --- | --- | --- | --- |
| rs9275312 | A/G | + | 6 | 32773706 | HLA-DQB1 | flanking_5’UTR | 9.25E-04 | 9.12E-04 |
| rs9275328 | C/T | + | 6 | 32774800 | HLA-DQB1 | flanking_5’UTR | 9.40E-04 | 9.14E-04 |
| rs660895 | A/G | + | 6 | 32685358 | HLA-DRB1 | flanking_5’UTR | 1.36E-03 | 1.48E-03 |
| rs7310659 | A/G | + | 12 | 14201016 | GRIN2B | flanking_5’UTR | 2.17E-03 | 2.09E-03 |
| rs13285154 | C/T | + | 9 | 31495016 | ACO1 | flanking_5’UTR | 4.03E-03 | 2.41E-03 |
| rs1542112 | C/T | + | 15 | 96669517 | FLJ39743 | flanking_3’UTR | 5.69E-03 | 6.74E-03 |
| rs3916765 | A/G | + | 6 | 32793528 | HLA-DQA2 | flanking_5’UTR | 7.26E-03 | 5.23E-03 |
| rs11079992 | A/G | + | 17 | 47572536 | CA10 | intron | 1.33E-02 | 2.99E-03 |
| rs6544127 | C/T | + | 2 | 38073146 | FAM82A | intron | 4.51E-02 | 8.35E-03 |
| rs655198 | A/G | + | 1 | 42098604 | HIVEP3 | intron | 1.09E-02 | 9.79E-03 |
| rs8085335 | A/G | + | 18 | 45363289 | SMAD2 | 3’ UTR | 1.81E-04 | 3.52E-04 |
| rs17085260 | A/C | + | 6 | 154639352 | PIP-3E | intron | 2.27E-04 | 7.54E-04 |
| rs324389 | C/T | + | 7 | 34744239 | NPSR1 | intron | 5.77E-05 | 1.45E-04 |
| rs10270663 | G/T | + | 7 | 34752923 | NPSR1 | intron | 3.56E-05 | 9.96E-05 |
| rs10267134 | A/G | + | 7 | 34769628 | NPSR1 | intron | 7.07E-05 | 1.03E-04 |
| rs10278663 | A/G | + | 7 | 34774996 | NPSR1 | intron | 7.07E-05 | 4.36E-04 |
|  |  |  |  |  |  |  |  |  |
