# Supplementary material for: Genome-Wide Association Study for Atopy and Allergic Rhinitis in a Singapore Chinese Population
Source: PLoS One. 2011 May 20;6(5):e19719. doi: 10.1371/journal.pone.0019719 (PMC3098846; doi:10.1371/journal.pone.0019719)
Supplement: Table S6 — Non-synonymous SNPs from the Illumina 550 k chip at a P-value<0.01. (DOCX) [file pone.0019719.s006.docx]

**Supplementary Table S6: Non-synonymous SNPs from the Illumina 550k chip at a p-value < 0.01**

| **SNP** | **Chr** | **Gene Symbol** | **Amino acid change** | **ID with Mouse** | **Phast Conservation** | **Atopy p-value** | **AR p-value** |
| --- | --- | --- | --- | --- | --- | --- | --- |
| rs891398 | 8 | CHRNA2 | T/A | 0.48 | 308 | 1.06E-03 | 3.98E-03 |
| rs11578336 | 1 | MAEL | S/A | 0.58 | 113 | 1.08E-03 | 2.12E-03 |
| rs1122326 | 17 | HSPB9 | Q/P | 0.51 | 25 | 1.39E-03 | 1.14E-03 |
| rs13273355 | 8 | C8orf48 | S/F | 0.57 | -1 | 1.54E-03 | 5.04E-03 |
| rs1919127 | 2 | C2orf16 | V/A | 0.67 | -1 | 1.79E-03 | 9.52E-04 |
| rs2273821 | 6 | MRPL18 | R/Q | 0.55 | -1 | 1.94E-03 | 1.04E-03 |
| rs1950902 | 14 | MTHFD1 | K/R | 0.67 | 327 | 2.05E-03 | 7.68E-03 |
| rs2298566 | 11 | SNX19 | L/R | 0.57 | 167 | 2.08E-03 | 1.87E-03 |
| rs897945 | 4 | THAP9 | L/F | -1 | 29 | 2.36E-03 | 4.17E-04 |
| rs625372 | 20 | SN | K/R | 0.56 | 27 | 2.52E-03 | 3.55E-04 |
| rs2499836 | 1 | OLFML2B | W/R | 0.52 | -1 | 2.71E-03 | 2.40E-03 |
| rs11241095 | 5 | WDR36 | I/V | 0.6 | 379 | 2.73E-03 | 1.75E-02 |
| rs2071307 | 7 | ELN | G/S | 0.56 | -1 | 2.89E-03 | 6.97E-03 |
| rs11646374 | 16 | FANCA | A/V | 0.61 | -1 | 3.62E-03 | 9.98E-03 |
| rs1126823 | 15 | AGC1 | Q/R | 0.59 | 110 | 3.69E-03 | 1.63E-03 |
| rs17121745 | 1 | WDR63 | T/A | 0.56 | 172 | 4.03E-03 | 4.12E-03 |
| rs2074158 | 17 | LGP2 | Q/R | 0.6 | -1 | 4.27E-03 | 2.56E-03 |
| rs6929137 | 6 | C6orf97 | V/I | 0.65 | 29 | 4.32E-03 | 1.58E-02 |
| rs3796543 | 4 | AASDH | K/R | 0.62 | -1 | 4.57E-03 | 1.39E-03 |
| rs1057190 | 12 | PUS7L | K/E | 0.64 | -1 | 4.59E-03 | 1.23E-02 |
| rs3747965 | 1 | DNTTIP2 | E/D | 0.66 | -1 | 4.70E-03 | 1.32E-03 |
| rs323345 | 8 | TEX15 | N/S | 0.68 | -1 | 4.95E-03 | 1.48E-02 |
| rs13021 | 14 | PNN | S/G | 0.77 | 2125 | 5.22E-03 | 1.67E-03 |
| rs7260180 | 19 | CEACAM20 | V/I | 0.65 | -1 | 5.24E-03 | 2.36E-02 |
| rs3748569 | 1 | RHBG | G/R | 0.57 | 104 | 5.78E-03 | 1.06E-02 |
| rs8080100 | 17 | HELZ | V/M | 0.54 | 171 | 6.06E-03 | 1.20E-02 |
| rs8113341 | 19 | LOC388503 | Q/R | 0.62 | -1 | 6.13E-03 | 1.50E-02 |
| rs4414223 | 11 | SNX19 | N/S | 0.61 | 151 | 6.63E-03 | 3.14E-03 |
| rs550510 | 17 | CALCOCO2 | G/E | 0.6 | 40 | 6.64E-03 | 1.41E-02 |
| rs1218762 | 16 | OR2C1 | G/S | 0.74 | -1 | 6.70E-03 | 2.16E-02 |
| rs2304053 | 5 | FAT2 | P/L | 0.69 | 44 | 7.02E-03 | 1.66E-02 |
| rs6000172 | 22 | APOL4 | S/L | 0.62 | -1 | 7.49E-03 | 2.53E-02 |
| rs12540919 | 7 | DFNA5 | V/M | 0.66 | -1 | 7.69E-03 | 9.59E-03 |
| rs2855430 | 6 | COL11A2 | L/P | 0.6 | 123 | 7.97E-03 | 1.36E-02 |
| rs1174657 | 1 | APOBEC4 | K/E | 0.67 | -1 | 8.13E-03 | 1.12E-02 |
| rs721917 | 10 | SFTPD | M/T | 0.58 | -1 | 8.27E-03 | 6.43E-03 |
| rs1468556 | 12 | GALNT8 | V/F | 0.5 | -1 | 8.59E-03 | 1.39E-02 |
| rs12419022 | 11 | OR5W2 | H/R | 0.65 | 46 | 8.67E-03 | 1.74E-02 |
| rs1174658 | 1 | APOBEC4 | F/S | 0.67 | -1 | 8.69E-03 | 1.21E-02 |
| rs2124147 | 3 | PLD1 | V/A | 0.59 | 327 | 8.72E-03 | 7.15E-03 |
| rs11230983 | 11 | OR5D13 | R/H | 0.65 | 53 | 9.25E-03 | 1.81E-02 |
| rs10911390 | 1 | APOBEC4 | V/M | 0.67 | -1 | 9.26E-03 | 6.47E-03 |
| rs297055 | 11 | OR5D14 | L/P | 0.76 | 91 | 9.85E-03 | 2.19E-02 |
| rs2472553 | 8 | CHRNA2 | T/I | 0.54 | 22 | 6.32E-04 | 1.80E-03 |
| rs3800544 | 6 | WDR27 | R/H | 0.52 | -1 | 1.52E-02 | 2.04E-03 |
| rs2227278 | 19 | ZBTB32 | R/S | 0.7 | -1 | 1.02E-02 | 2.30E-03 |
| rs1801033 | 5 | C6 | A/E | 0.61 | 144 | 1.23E-02 | 3.29E-03 |
| rs2306595 | 17 | MYOHD1 | N/S | 0.51 | 155 | 1.02E-02 | 3.53E-03 |
| rs2276932 | 4 | ARHGAP10 | M/V | 0.61 | 33 | 1.22E-02 | 3.56E-03 |
| rs273957 | 7 | CREB3L2 | V/I | 0.57 | -1 | 6.30E-04 | 3.92E-03 |
| rs2306393 | 12 | MDM1 | R/H | 0.57 | -1 | 2.75E-02 | 4.43E-03 |
| rs962976 | 12 | MDM1 | T/I | 0.61 | -1 | 2.93E-02 | 4.56E-03 |
| rs12026290 | 1 | PHC2 | V/M | 0.62 | -1 | 1.78E-02 | 4.58E-03 |
| rs1815811 | 11 | PDZD3 | R/Q | 0.66 | -1 | 1.59E-02 | 4.72E-03 |
| rs2075820 | 7 | CARD4 | E/K | 0.77 | 265 | 3.19E-02 | 5.11E-03 |
| rs3765148 | 19 | DHDH | G/R | 0.58 | -1 | 1.15E-02 | 5.19E-03 |
| rs2072355 | 12 | AKAP3 | G/E | 0.71 | -1 | 2.35E-02 | 5.68E-03 |
| rs1395 | 2 | SLC5A6 | S/F | 0.57 | -1 | 1.56E-02 | 6.00E-03 |
| rs1260326 | 2 | GCKR | L/P | 0.64 | -1 | 1.57E-02 | 6.31E-03 |
| rs6025606 | 20 | CTCFL | T/A | 0.57 | -1 | 1.37E-02 | 7.67E-03 |
| rs10079250 | 5 | CSF1R | H/R | 0.57 | -1 | 2.79E-02 | 8.51E-03 |
| rs3744137 | 17 | M-RIP | P/Q | 0.53 | -1 | 2.11E-02 | 9.28E-03 |
| rs2904979 | 11 | FLJ16331 | T/I | -1 | -1 | 6.34E-03 | 2.40E-03 |
| rs1945196 | 11 | OR5M11 | P/H | 0.7 | -1 | 7.17E-03 | 1.76E-02 |
| rs7246479 | 19 | BRSK1 | L/F | 0.67 | -1 | 9.93E-03 | 9.45E-03 |
| rs486557 | 1 | TMEM51 | R/H | 0.63 | -1 | 1.17E-02 | 4.73E-03 |
| rs7279142 | 21 | TIAM1 | A/T | -1 | -1 | 1.05E-02 | 9.16E-03 |
| rs4948550 | 10 | PHYHIPL | S/P | 0.63 | 378 | 6.40E-03 | 3.65E-03 |
| rs4665809 | 2 | RAB10 | F/L | 0.71 | -1 | 3.14E-02 | 6.00E-03 |
| rs3765966 | 1 | CA6 | R/L | -1 | -1 | 2.47E-03 | 1.47E-02 |
| rs2295547 | 20 | C20orf116 | F/L | 0.63 | -1 | 1.33E-02 | 2.88E-03 |
| rs2286428 | 7 | ZP3 | G/R | 0.56 | -1 | 6.19E-02 | 8.49E-03 |
