# Supplementary material for: Genome-Wide Association Study for Atopy and Allergic Rhinitis in a Singapore Chinese Population
Source: PLoS One. 2011 May 20;6(5):e19719. doi: 10.1371/journal.pone.0019719 (PMC3098846; doi:10.1371/journal.pone.0019719)
Supplement: Table S7 — Putative effect of the non-synonymous SNPs as predicted by SIFT. (DOCX) [file pone.0019719.s007.docx]

**Supplementary Table S7: Putative effect of non-synonymous SNPs as predicted by SIFT^**

| **SNP** | **Amino acid (a.a) change** | **a.a** | **Using orthologues in the**  **protein alignment** | | | **Using orthologues in the**  **protein alignment** | | |
| --- | --- | --- | --- | --- | --- | --- | --- | --- |
|  |  |  | **Prediction** | **Score** | **Median IC** | **Prediction** | **Score** | **Median info** |
| rs891398 | T125A | T | TOLERATED | 0.53 | 2.77 | TOLERATED | 0.16 | 3.68 |
|  |  | A | TOLERATED | 1 |  | TOLERATED | 1 |  |
| rs11578336 | S41A | S | TOLERATED | 0.35 | 2.75 | TOLERATED | 0.23 | 2.85 |
|  |  | A | TOLERATED | 0.85 |  | TOLERATED | 1 |  |
| rs1122326 | Q2P | Q | TOLERATED | 0.8 | 2.77 | TOLERATED | 1 | 2.92 |
|  |  | P | DAMAGING | 0.03 |  | DAMAGING | 0.04 |  |
| rs1919127 | V685A | V | TOLERATED | 1 | 4.32 | TOLERATED | 1 | 3.32 |
|  |  | A | TOLERATED | 0.08 |  | TOLERATED | 0.32 |  |
| rs1950902 | R134K | R | TOLERATED | 0.85 | 3.31 | TOLERATED | 0.98 | 3.56 |
|  |  | K | TOLERATED | 0.82 |  | TOLERATED | 1 |  |
| rs2298566 | L878R | L | TOLERATED | 0.3 | 2.84 | TOLERATED | 0.07 | 3.1 |
|  |  | R | TOLERATED | 1 |  | TOLERATED | 1 |  |
| rs897945 | L299F | L | TOLERATED | 0.6 | 2.79 | TOLERATED | 1 | 2.77 |
|  |  | F | TOLERATED | 1 |  | TOLERATED | 0.19 |  |
| rs2499836 | W470R | W | TOLERATED | 0.12 | 3.7 | DAMAGING | 0.03 | 2.94 |
|  |  | R | TOLERATED | 0.68 |  | TOLERATED | 0.2 |  |
| rs11241095 | I264V | I | TOLERATED | 0.93 | 2.81 | TOLERATED | 0.95 | 2.82 |
|  |  | V | TOLERATED | 1 |  | TOLERATED | 1 |  |
| rs2071307 | G422S | G | TOLERATED | 1 | 2.81 | TOLERATED | 0.58 | 2.99 |
|  |  | S | TOLERATED | 0.52 |  | TOLERATED | 0.48 |  |
| rs11646374 | A412V | A | TOLERATED | 0.94 | 2.76 | TOLERATED | 1 | 2.94 |
|  |  | V | TOLERATED | 0.34 |  | TOLERATED | 0.08 |  |
| rs17121745 | T674A | T | TOLERATED | 0.43 | 2.83 | TOLERATED | 0.44 | 2.8 |
|  |  | A | TOLERATED | 0.58 |  | TOLERATED | 0.62 |  |
| rs2074158 | Q425R | Q | TOLERATED | 1 | 2.79 | TOLERATED | 1 | 2.9 |
|  |  | R | TOLERATED | 0.46 |  | TOLERATED | 0.64 |  |
| rs6929137 | V604I | V | TOLERATED | 1 | 2.9 | TOLERATED | 1 | 2.89 |
|  |  | I | TOLERATED | 0.48 |  | TOLERATED | 0.47 |  |
| rs3796543 | K368R | K | TOLERATED | 1 | 2.81 | TOLERATED | 1 | 2.79 |
|  |  | R | TOLERATED | 0.72 |  | TOLERATED | 0.81 |  |
| rs1057190 | K264E | K | TOLERATED | 0.38 | 2.91 | TOLERATED | 0.3 | 2.82 |
|  |  | E | TOLERATED | 1 |  | TOLERATED | 1 |  |
| rs3747965 | E309D | E | TOLERATED | 1 | 2.82 | TOLERATED | 1 | 2.91 |
|  |  | D | TOLERATED | 0.26 |  | TOLERATED | 0.25 |  |
| rs13021 | S671G | S | TOLERATED | 1 | 3.25 | TOLERATED | 1 | 3.63 |
|  |  | G | TOLERATED | 0.64 |  | TOLERATED | 0.13 |  |
| rs7260180 | V108I | V | TOLERATED | 0.92 | 2.83 | TOLERATED | 0.18 | 2.8 |
|  |  | I | TOLERATED | 1 |  | TOLERATED | 0.27 |  |
| rs3748569 | G315R | G | TOLERATED | 1 | 2.75 | TOLERATED | 1 | 3.68 |
|  |  | R | DAMAGING | 0 |  | DAMAGING | 0.01 | *Warning! Low confidence.** |
| rs8080100 | V74M | V | TOLERATED | 0.06 | 3 | TOLERATED | 0.05 | 2.98 |
|  |  | M | DAMAGING | 0.02 |  | DAMAGING | 0.01 |  |
| rs8113341 | Q172R | Q | TOLERATED | 1 | 2.83 | TOLERATED | 1 | 2.98 |
|  |  | R | TOLERATED | 0.36 |  | TOLERATED | 0.19 |  |
| rs4414223 | N753S | N | TOLERATED | 1 | 3.33 | TOLERATED | 1 | 3.03 |
|  |  | S | TOLERATED | 0.6 |  | TOLERATED | 0.54 |  |
| rs550510 | G140E | G | TOLERATED | 0.82 | 2.8 | TOLERATED | 0.49 | 2.93 |
|  |  | E | TOLERATED | 0.67 |  | TOLERATED | 1 |  |
| rs1218762 | G16S | G | TOLERATED | 1 | 2.84 | TOLERATED | 1 | 3.27 |
|  |  | S | TOLERATED | 0.12 |  | DAMAGING | 0.04 | *Warning! Low confidence.** |
| rs2304053 | P1164L | P | TOLERATED | 0.71 | 2.89 | TOLERATED | 1 | 2.84 |
|  |  | L | TOLERATED | 0.21 |  | TOLERATED | 0.13 |  |
| rs12540919 | V207M | V | TOLERATED | 1 | 2.91 | TOLERATED | 1 | 2.94 |
|  |  | M | TOLERATED | 0.06 |  | DAMAGING | 0.03 |  |
| rs2855430 | P787L | P | TOLERATED | 0.43 | 2.92 | TOLERATED | 1 | 4.32 |
|  |  | L | TOLERATED | 0.38 |  | DAMAGING | 0 | *Warning! Low confidence.** |
| rs1174657 | K331E | K | TOLERATED | 1 | 3.59 | TOLERATED | 1 | 3.6 |
|  |  | E | TOLERATED | 0.38 |  | TOLERATED | 0.23 |  |
| rs721917 | M31T | M | TOLERATED | 0.16 | 2.89 | TOLERATED | 0.22 | 3.63 |
|  |  | T | TOLERATED | 0.57 |  | TOLERATED | 0.19 |  |
| rs1468556 | V515F | V | TOLERATED | 0.31 | 2.81 | TOLERATED | 0.61 | 2.94 |
|  |  | F | TOLERATED | 0.06 |  | TOLERATED | 0.11 |  |
| rs12419022 | H65R | H | TOLERATED | 0.62 | 2.79 | TOLERATED | 1 | 3.83 |
|  |  | R | DAMAGING | 0.02 |  | DAMAGING | 0 | *Warning! Low confidence.** |
| rs1174658 | F271S | F | TOLERATED | 0.73 | 3.59 | TOLERATED | 0.72 | 3.6 |
|  |  | S | TOLERATED | 0.93 |  | TOLERATED | 0.97 |  |
| rs11230983 | R124H | R | TOLERATED | 1 | 2.79 | TOLERATED | 1 | 3.47 |
|  |  | H | TOLERATED | 0.11 |  | TOLERATED | 0.09 |  |
| rs10911390 | V345M | V | TOLERATED | 1 | 3.59 | TOLERATED | 1 | 3.6 |
|  |  | M | DAMAGING | 0.02 | *Warning!Low confidence.** | DAMAGING | 0.03 | *Warning! Low confidence.** |
| rs297055 | L290P | L | TOLERATED | 1 | 2.87 | TOLERATED | 1 | 3.63 |
|  |  | P | DAMAGING | 0.01 |  | DAMAGING | 0.01 | *Warning! Low confidence.** |
| rs2472553 | T22I | T | TOLERATED | 0.44 | 2.76 | TOLERATED | 0.12 | 4.32 |
|  |  | I | TOLERATED | 0.44 |  | TOLERATED | 0.49 |  |
| rs3800544 | R467H | R | TOLERATED | 0.77 | 2.79 | TOLERATED | 0.79 | 2.89 |
|  |  | H | TOLERATED | 0.17 |  | TOLERATED | 0.4 |  |
| rs2227278 | R174S | R | TOLERATED | 0.32 | 3.08 | TOLERATED | 0.18 | 2.97 |
|  |  | S | TOLERATED | 1 |  | TOLERATED | 0.64 |  |
| rs1801033 | A119E | A | TOLERATED | 0.4 | 2.77 | TOLERATED | 0.36 | 2.8 |
|  |  | E | TOLERATED | 1 |  | TOLERATED | 1 |  |
| rs2306595 | N176S | N | TOLERATED | 1 | 2.77 | TOLERATED | 1 | 2.92 |
|  |  | S | TOLERATED | 0.16 |  | DAMAGING | 0 |  |
| rs2276932 | M684V | M | TOLERATED | 0.92 | 2.91 | TOLERATED | 0.17 | 3.35 |
|  |  | V | TOLERATED | 1 |  | TOLERATED | 0.29 |  |
| rs2306393 | R489H | R | TOLERATED | 0.73 | 2.83 | TOLERATED | 0.83 | 2.97 |
|  |  | H | TOLERATED | 0.6 |  | TOLERATED | 0.13 |  |
| rs962976 | T103I | T | TOLERATED | 1 | 3.06 | TOLERATED | 1 | 3.05 |
|  |  | I | TOLERATED | 0.1 |  | TOLERATED | 0.12 |  |
| rs12026290 | V475M | V | TOLERATED | 1 | 3.03 | TOLERATED | 0.22 | 2.97 |
|  |  | M | TOLERATED | 0.15 |  | TOLERATED | 0.06 |  |
| rs1815811 | Q358R | Q | TOLERATED | 0.4 | 3.34 | TOLERATED | 0.49 | 3.19 |
|  |  | R | TOLERATED | 0.44 |  | TOLERATED | 0.72 |  |
| rs2075820 | E266K | E | TOLERATED | 0.97 | 2.84 | TOLERATED | 0.64 | 3.05 |
|  |  | K | TOLERATED | 0.2 |  | DAMAGING | 0.01 |  |
| rs3765148 | G282R | G | TOLERATED | 0.48 | 2.79 | TOLERATED | 0.53 | 2.79 |
|  |  | R | TOLERATED | 0.05 |  | DAMAGING | 0.03 |  |
| rs2072355 | G118E | G | TOLERATED | 0.41 | 3.08 | TOLERATED | 0.66 | 2.8 |
|  |  | E | TOLERATED | 0.9 |  | TOLERATED | 1 |  |
| rs1395 | F481S | F | TOLERATED | 0.69 | 2.78 | DAMAGING | 0.04 | 2.96 |
|  |  | S | TOLERATED | 0.56 |  | TOLERATED | 1 |  |
| rs1260326 | P446L | P | TOLERATED | 0.17 | 2.86 | TOLERATED | 1 | 3.09 |
|  |  | L | TOLERATED | 1 |  | DAMAGING | 0 |  |
| rs6025606 | T177A | T | TOLERATED | 1 | 3.12 | TOLERATED | 1 | 3.14 |
|  |  | A | TOLERATED | 0.35 |  | TOLERATED | 0.26 |  |
| rs10079250 | H362R | H | TOLERATED | 0.1 | 2.78 | TOLERATED | 0.08 | 2.8 |
|  |  | R | DAMAGING | 0.01 |  | DAMAGING | 0 |  |
| rs3744137 | P327Q | P | TOLERATED | 0.34 | 2.82 | TOLERATED | 0.23 | 2.91 |
|  |  | Q | TOLERATED | 0.59 |  | TOLERATED | 0.37 |  |
| rs7279142 | A61T | A | TOLERATED | 0.74 | 3.21 | TOLERATED | 0.14 | 3.55 |
|  |  | T | TOLERATED | 0.9 |  | TOLERATED | 0.37 |  |
| rs4948550 | S943P | S | TOLERATED | 0.74 | 3 | TOLERATED | 0.48 | 2.91 |
|  |  | P | TOLERATED | 1 |  | TOLERATED | 0.27 |  |
| rs4665809 | F41L | F | TOLERATED | 1 | 2.77 | TOLERATED | 0.75 | 4.32 |
|  |  | L | TOLERATED | 0.08 |  | TOLERATED | 1 |  |

^Predicting the effects of coding non-synonymous variants on protein function using the SIFT algorithm, Nature Protocols 4, - 1073 - 1081 (2009) Available at http://sift.jcvi.org/. Accessed 2010 Nov 16

* Indicates a warning because the prediction has low confidence as the protein alignment does not have enough sequence diversity. This may be due to the fact that the position artificially ap-pears to be conserved, an amino acid may incorrectly predict to be damaging.
