# Supplementary material for: Genome-Wide Association Study for Atopy and Allergic Rhinitis in a Singapore Chinese Population
Source: PLoS One. 2011 May 20;6(5):e19719. doi: 10.1371/journal.pone.0019719 (PMC3098846; doi:10.1371/journal.pone.0019719)
Supplement: Table S8 — Putative function of non-synonymous SNPs. (DOCX) [file pone.0019719.s008.docx]

**Supplementary Table S8: Putative function of non-synonymous SNPs as predicted by Tabor *et al*., [1] and Yuan *et al*., [2]**

| **SNPID**  **(rs id)** | **Possible Functional Effects** | **Lower Risk** | **Upper Risk** | | **Gene Symbol** | **Different** | **Different** |
| --- | --- | --- | --- | --- | --- | --- | --- |
|  |  |  |  |  |  | **ESEFINDER** | **RESCUEESE** |
| rs2306393 | Missense(non-conservative) | 3 | 4 | MDM1 | | FALSE | FALSE |
| rs297055 | Missense(non-conservative) | 3 | 4 | OR5D14 | | FALSE | FALSE |
| rs962976 | Missense(non-conservative) | 3 | 4 | MDM1 | | FALSE | FALSE |
| rs1122326 | Missense(non-conservative);Splicing regulation | 3 | 4 | HSPB9 | | TRUE | TRUE |
| rs12419022 | Missense(non-conservative);Splicing regulation | 3 | 4 | OR5W2 | | FALSE | TRUE |
| rs13273355 | Missense(non-conservative);Splicing regulation | 3 | 4 | C8orf48 | | TRUE | TRUE |
| rs2276932 | Missense(non-conservative);Splicing regulation | 3 | 4 | ARHGAP10 | | TRUE | FALSE |
| rs2298566 | Missense(non-conservative);Splicing regulation | 3 | 4 | SNX19 | | TRUE | TRUE |
| rs2306595 | Missense(non-conservative);Splicing regulation | 3 | 4 | MYO19 | | TRUE | FALSE |
| rs3744137 | Missense(non-conservative);Splicing regulation | 3 | 4 | MPRIP | | TRUE | FALSE |
| rs8113341 | Missense(non-conservative);Splicing regulation | 3 | 4 | C3P1 | | TRUE | TRUE |
| rs11241095 | Missense(conservative) | 2 | 3 | WDR36 | | FALSE | FALSE |
| rs11646374 | Missense(conservative) | 2 | 3 | FANCA | | FALSE | FALSE |
| rs13021 | Missense(conservative) | 2 | 3 | PNN | | FALSE | FALSE |
| rs1945196 | Missense(conservative) | 2 | 3 | OR5M5P | | FALSE | FALSE |
| rs1950902 | Missense(conservative) | 2 | 3 | MTHFD1 | | FALSE | FALSE |
| rs2072355 | Missense(conservative) | 2 | 3 | AKAP3 | | FALSE | FALSE |
| rs2227278 | Missense(conservative) | 2 | 3 | ZBTB32 | | FALSE | FALSE |
| rs2472553 | Missense(conservative) | 2 | 3 | CHRNA2 | | FALSE | FALSE |
| rs2904979 | Missense(conservative) | 2 | 3 | SLC22A20 | | FALSE | FALSE |
| rs3765148 | Missense(conservative) | 2 | 3 | DHDH | | FALSE | FALSE |
| rs8080100 | Missense(conservative) | 2 | 3 | HELZ | | FALSE | FALSE |
| rs891398 | Missense(conservative) | 2 | 3 | CHRNA2 | | FALSE | FALSE |
| rs1057190 | Missense(conservative);Splicing regulation | 2 | 3 | PUS7L | | FALSE | TRUE |
| rs10911390 | Missense(conservative);Splicing regulation | 2 | 3 | APOBEC4 | | TRUE | FALSE |
| rs11230983 | Missense(conservative);Splicing regulation | 2 | 3 | OR5D13 | | TRUE | FALSE |
| rs1126823 | Missense(conservative);Splicing regulation | 2 | 3 | ACAN | | TRUE | FALSE |
| rs11578336 | Missense(conservative);Splicing regulation | 2 | 3 | MAEL | | TRUE | FALSE |
| rs1174657 | Missense(conservative);Splicing regulation | 2 | 3 | APOBEC4 | | TRUE | FALSE |
| rs1174658 | Missense(conservative);Splicing regulation | 2 | 3 | APOBEC4 | | TRUE | TRUE |
| rs12026290 | Missense(conservative);Splicing regulation | 2 | 3 | PHC2 | | TRUE | FALSE |
| rs1218762 | Missense(conservative);Splicing regulation | 2 | 3 | OR2C1 | | TRUE | TRUE |
| rs12540919 | Missense(conservative);Splicing regulation | 2 | 3 | DFNA5 | | TRUE | FALSE |
| rs1395 | Missense(conservative);Splicing regulation | 2 | 3 | SLC5A6 | | TRUE | TRUE |
| rs1468556 | Missense(conservative);Splicing regulation | 2 | 3 | GALNT8 | | TRUE | FALSE |
| rs17121745 | Missense(conservative);Splicing regulation | 2 | 3 | WDR63 | | TRUE | TRUE |
| rs1801033 | Missense(conservative);Splicing regulation | 2 | 3 | C6 | | TRUE | FALSE |
| rs1815811 | Missense(conservative);Splicing regulation | 2 | 3 | PDZD3 | | TRUE | FALSE |
| rs1919127 | Missense(conservative);Splicing regulation | 2 | 3 | C2orf16 | | TRUE | FALSE |
| rs2071307 | Missense(conservative);Splicing regulation | 2 | 3 | ELN | | TRUE | FALSE |
| rs2074158 | Missense(conservative);Splicing regulation | 2 | 3 | DHX58 | | TRUE | TRUE |
| rs2075820 | Missense(conservative);Splicing regulation | 2 | 3 | NOD1 | | TRUE | FALSE |
| rs2124147 | Missense(conservative);Splicing regulation | 2 | 3 | PLD1 | | TRUE | TRUE |
| rs2286428 | Missense(conservative);Splicing regulation | 2 | 3 | ZP3 | | TRUE | FALSE |
| rs2295547 | Missense(conservative);Splicing regulation | 2 | 3 | DDRGK1 | | TRUE | FALSE |
| rs2304053 | Missense(conservative);Splicing regulation | 2 | 3 | FAT2 | | TRUE | FALSE |
| rs2499836 | Missense(conservative);Splicing regulation | 2 | 3 | OLFML2B | | TRUE | FALSE |
| rs273957 | Missense(conservative);Splicing regulation | 2 | 3 | CREB3L2 | | TRUE | TRUE |
| rs323345 | Missense(conservative);Splicing regulation | 2 | 3 | TEX15 | | TRUE | FALSE |
| rs3747965 | Missense(conservative);Splicing regulation | 2 | 3 | DNTTIP2 | | FALSE | TRUE |
| rs3748569 | Missense(conservative);Splicing regulation | 2 | 3 | RHBG | | TRUE | FALSE |
| rs3765966 | Missense(conservative);Splicing regulation | 2 | 3 | CA6 | | TRUE | TRUE |
| rs4414223 | Missense(conservative);Splicing regulation | 2 | 3 | SNX19 | | TRUE | FALSE |
| rs486557 | Missense(conservative);Splicing regulation | 2 | 3 | FHAD1 | | TRUE | FALSE |
| rs4948550 | Missense(conservative);Splicing regulation | 2 | 3 | BICC1 | | TRUE | FALSE |
| rs6000172 | Missense(conservative);Splicing regulation | 2 | 3 | APOL4 | | TRUE | FALSE |
| rs6025606 | Missense(conservative);Splicing regulation | 2 | 3 | CTCFL | | TRUE | TRUE |
| rs625372 | Missense(conservative);Splicing regulation | 2 | 3 | SIGLEC1 | | TRUE | FALSE |
| rs6929137 | Missense(conservative);Splicing regulation | 2 | 3 | C6orf97 | | TRUE | FALSE |
| rs721917 | Missense(conservative);Splicing regulation | 2 | 3 | SFTPD | | FALSE | TRUE |
| rs7246479 | Missense(conservative);Splicing regulation | 2 | 3 | TMEM150B | | FALSE | TRUE |
| rs7260180 | Missense(conservative);Splicing regulation | 2 | 3 | CEACAM20 | | TRUE | FALSE |
| rs7279142 | Missense(conservative);Splicing regulation | 2 | 3 | KRTAP19-8 | | TRUE | FALSE |
| rs897945 | Missense(conservative);Splicing regulation | 2 | 3 | THAP9 | | FALSE | TRUE |

(1) Tabor et al. (2002) Opinion: candidate-gene approaches for studying complex genetic traits: practical considerations Nature Rev. Genet., 3, 391–397. doi: 10.1038/nrg796

(2) FASTSNP: an always up-to-date and extendable service for SNP function analysis and prioritization. Nucleic Acids Res., 2006, 34, Web Server issue, W635-41
