# Supplementary material for: Genome-Wide Association Study for Atopy and Allergic Rhinitis in a Singapore Chinese Population
Source: PLoS One. 2011 May 20;6(5):e19719. doi: 10.1371/journal.pone.0019719 (PMC3098846; doi:10.1371/journal.pone.0019719)
Supplement: Table S9 — Power calculation for one stage design to detect association at genome wide significance (P<5*10−8). (DOCX) [file pone.0019719.s009.docx]

| **Phenotype** | **Case/Control counts** | **Disease allele frequency** | **Odds Ratio** | **Power** |
| --- | --- | --- | --- | --- |
| **Atopy** | 2838/997 | 0.05 | 1.5 | 14% |
|  |  |  | 1.8 | 90% |
|  |  | 0.10 | 1.5 | 68% |
|  |  |  | 1.8 | 100% |
|  |  | 0.15 | 1.5 | 93% |
|  |  |  | 1.8 | 100% |
| **Allergic Rhinitis** | 1132/997 | 0.05 | 1.5 | 50% |
|  |  |  | 1.8 | 100 |
|  |  | 0.10 | 1.5 | 95% |
|  |  |  | 1.8 | 95% |
|  |  | 0.15 | 1.5 | 100% |
|  |  |  | 1.8 | 100% |

**Supplementary Table S9: Power Calculation for the Atopy and Allergic Rhinitis phenotypes at a prevalence of 25% for the Two-stage Case Control Association Study**
