# Supplementary figures and images for: Genome-Wide Association Study for Atopy and Allergic Rhinitis in a Singapore Chinese Population
Source: PLoS One. 2011 May 20;6(5):e19719. doi: 10.1371/journal.pone.0019719 (PMC3098846; doi:10.1371/journal.pone.0019719)

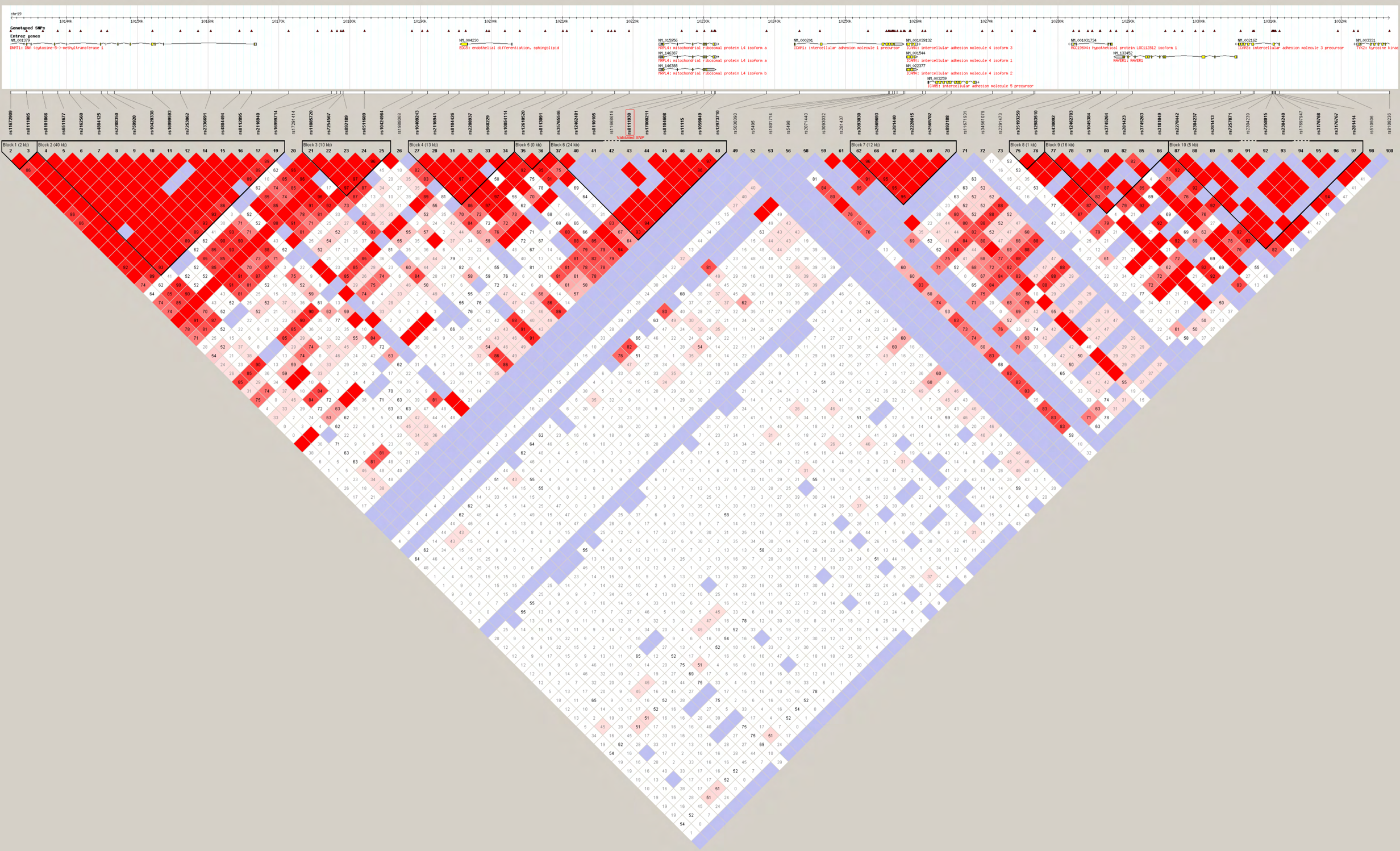

Supplement: Figure S2 — Genomic organization of a 200-kb region of chromosome 19p13.2 containing rs8111930 with linkage disequilibrium information. (PDF) [file pone.0019719.s011.pdf]

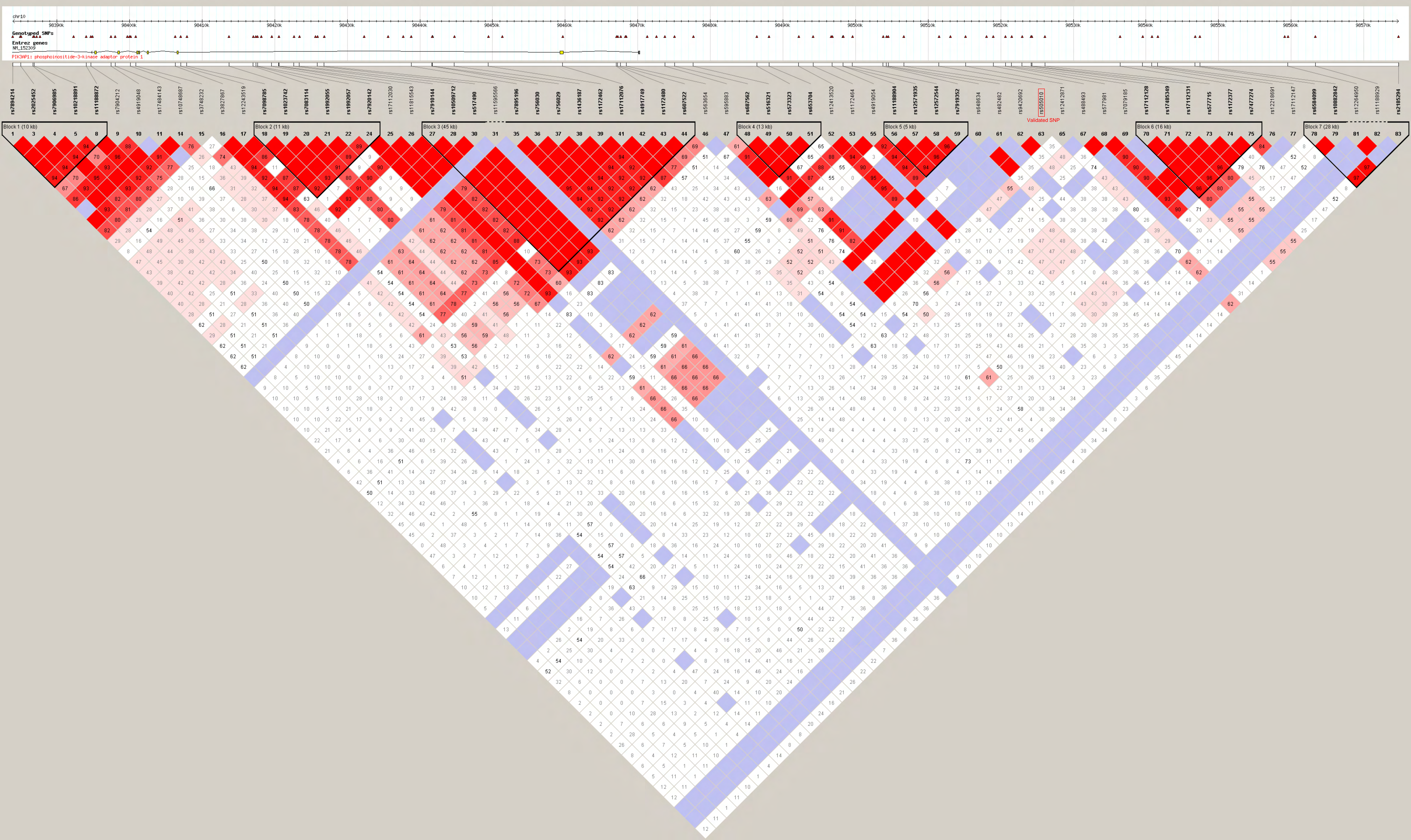

Supplement: Figure S3 — Genomic organization of a 200-kb region of chromosome 10q24.1 containing rs505010 with linkage disequilibrium information. (PDF) [file pone.0019719.s012.pdf]
