# Supplementary material for: Genome-Wide Association Study for Atopy and Allergic Rhinitis in a Singapore Chinese Population
Source: PLoS One. 2011 May 20;6(5):e19719. doi: 10.1371/journal.pone.0019719 (PMC3098846; doi:10.1371/journal.pone.0019719)
Supplement: Figure S6 — Quantile-quantile (Q-Q) plots of the observed P values versus the expected values from P value of association for the previously reported asthma SNPs tested for (A) Atopy and (B) Allergic Rhinitis. (DOC) [file pone.0019719.s015.doc]

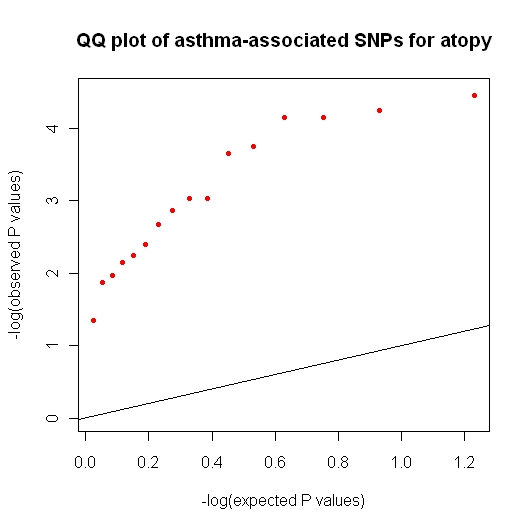

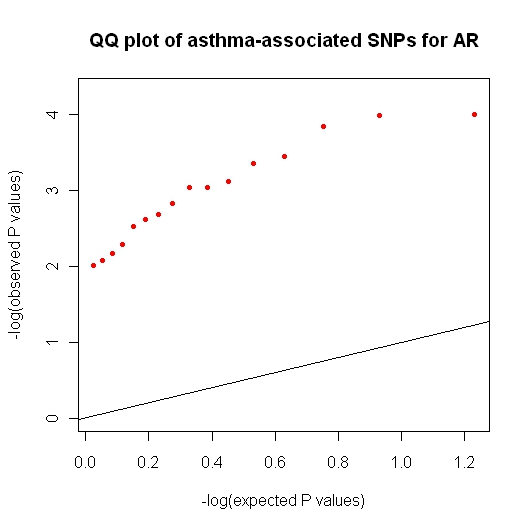


**Supplementary Figure 6**: Quantile-quantile (Q-Q) plots of the observed *P* values versus the expected values from *P* value of association for the previously reported asthma SNPs tested for (A) Atopy and (B) Allergic Rhinitis
